# Targeting early proximal-rod component substrate FlgB to FlhB for flagellar-type III secretion in *Salmonella*

**DOI:** 10.1371/journal.pgen.1010313

**Published:** 2022-07-12

**Authors:** Daofeng Qu, Mengxue Jiang, Calder Duffin, Kelly T. Hughes, Fabienne F. V. Chevance

**Affiliations:** 1 Key Laboratory of Food Quality and Safety, School of Food Science and Biotechnology, Zhejiang Gongshang University, Hangzhou, China; 2 School of Biological Sciences, University of Utah, Salt Lake City, Utah, United States of America; Universidad de Sevilla, SPAIN

## Abstract

The *Salmonella* flagellar secretion apparatus is a member of the type III secretion (T3S) family of export systems in bacteria. After completion of the flagellar motor structure, the hook-basal body (HBB), the flagellar T3S system undergoes a switch from early to late substrate secretion, which results in the expression and assembly of the external, filament propeller-like structure. In order to characterize early substrate secretion-signals in the flagellar T3S system, the FlgB, and FlgC components of the flagellar rod, which acts as the drive-shaft within the HBB, were subject to deletion mutagenesis to identify regions of these proteins that were important for secretion. The β-lactamase protein lacking its Sec-dependent secretion signal (Bla) was fused to the C-terminus of FlgB and FlgC and used as a reporter to select for and quantify the secretion of FlgB and FlgC into the periplasm. Secretion of Bla into the periplasm confers resistance to ampicillin. In-frame deletions of amino acids 9 through 18 and amino acids 39 through 58 of FlgB decreased FlgB secretion levels while deleting amino acid 6 through 14 diminished FlgC secretion levels. Further PCR-directed mutagenesis indicated that amino acid F45 of FlgB was critical for secretion. Single amino acid mutagenesis revealed that all amino acid substitutions at F45 of FlgB position impaired rod assembly, which was due to a defect of FlgB secretion. An equivalent F49 position in FlgC was essential for assembly but not for secretion. This study also revealed that a hydrophobic patch in the cleaved C-terminal domain of FlhB is critical for recognition of FlgB at F45.

## Author summary

Type III secretion (T3S) is the means by which proteins are secreted from the bacterial cytoplasm to build flagella for motility and injectisome structures that facilitate pathogenesis. T3S is the only secretion system known to date that undergoes a secretion-specificity switch. For the assembly of the bacterial flagellum, the T3S system initially secretes early substrates to build the hook-basal body (HBB), which is the main component that makes up the flagellar motor. Upon HBB completion, the flagellar T3S system becomes specific for late substrates, which make up the long external filament that acts as the propeller of the motility organelle. This work identifies important sites of interaction between an early substrate, FlgB and a target site at the cytoplasmic base of T3S apparatus. A second early substrate, FlgC, lacks the targeting interaction found for FlgB suggesting a mechanism that distinguishes early substrates, and may indicate an order to early substrate secretion to facilitate the order of protein subunit assembly for the flagellum.

## Introduction

Type III secretion (T3S) is the mechanism for protein secretion to build the bacterial flagellum and the virulence-associated injectisome structures used by Gram-negative plant and animal pathogens to inject effector proteins into host cells to facilitate pathogenesis [1–3]. T3S has evolved to be highly efficient exporting substrates at rates of thousands of amino acids per second [4,5]. The high rate of secretion necessitates that protein secretion be directly coupled to the proton motive force [6,7].

**Fig 1:**
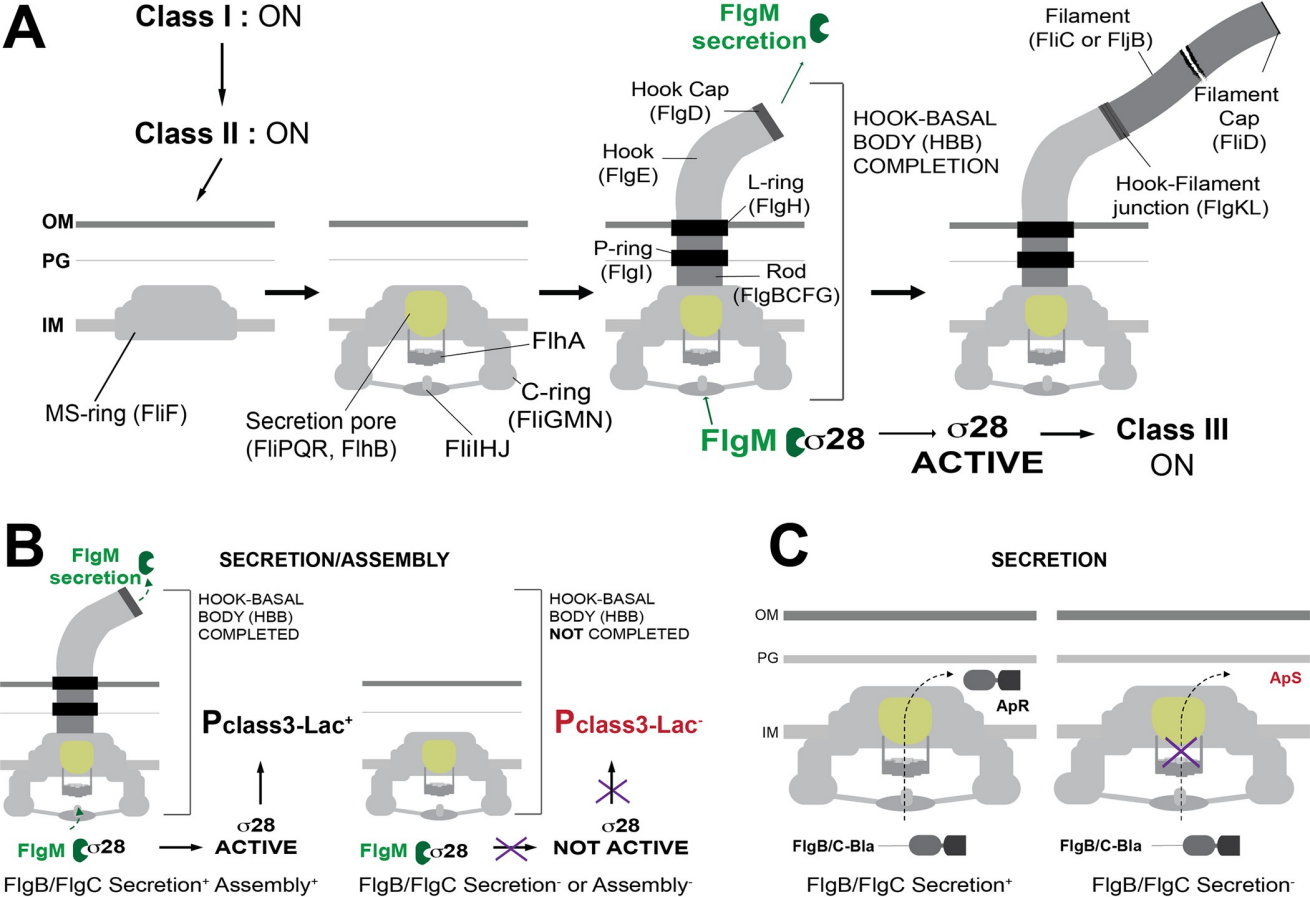
Flagellar assembly steps and assays to measure secretion/assembly of early rod substrates. **A.** Flagellum assembly initiates with MS-ring assembly in the inner membrane (IM). The C-ring rotor assemble below the MS-ring. Within the MS-ring the core secretion apparatus forms, which includes FliP_5_-FliQ_4_-FliR-FlhB where FliP_5_ is the actual internal pore through which subunits are secreted. Beneath the FliP_5_Q_4_RFlhB core is a large nonameric ring structure formed from 9 identical subunits of FlhA. The N-terminal 330 residues of FlhA includes 8 predicted transmembrane segments followed by a 33 amino acid linker region followed by a large 328 residue C-terminal domain that folds into 4 distinct subdomains, which will crystallize into a nonameric structure for which the structure has been solved. FlhA_9_ exists in two forms of the C-terminal nonamer, a closed and open form. The closed form allows recognition of early secretion substrates required for the structure and assembly of the rod-hook components of the HBB. The open form allows recognition of late secretion substrates FlgK, FlgL, FliD, FlgM and the filament subunits FliC or FljB. Beneath FlhA is the FliH_2_I_6_J ATPase, which facilitates substrate delivery to FlhA_9_ and hydrolyzes ATP to release and unfold substrates prior to secretion. HBB completion is coupled to a secretion-specificity switch where early rod-hook substrates are no longer recognized and the secretion apparatus is specific for late secretion-substrates. Once the hook-basal body (HBB) structure is completed, FlgM is secreted and σ^28^ is free to direct transcription from class 3 flagellar promoters to produce hook-filament junction proteins, the filament cap and filament proteins FliC or FljB, which are alternatively expressed. In this way, flagellar class 3 promoter transcription is coupled to HBB completion **B.** Use of a *lac* operon reporter to a flagellar class 3 promoter provides a genetic screen for defects in HBB assembly. Any mutant that is defective in HBB assembly does not undergo the secretion-specificity switch. FlgM remains in the cytoplasm bound to σ^28^ and class 3 promoter transcription is inhibited. The MudJ transposable element is a *lac* operon reporter, which when fused to a class 3 flagellar promoter, such as a *fljB*::MudJ fusion, provides an indicator for defects in secretion and/or assembly of FlgB or FlgC proximal rod subunits [21]. Mutants in *flgB* or *flgC* that are defective in secretion and/or assembly produce a Lac^-^ phenotype in the presence of the *fljB*::MudJ reporter. **C**. Fusion of FlgB or FlgC to β-lactamase, lacking its Sec-dependent secretion signal (Bla), provides a selection/screen for secretion of FlgB or FlgC into the periplasm. Bla must be secreted into the periplasm to fold into an active conformation to confer ampicillin resistance (Ap^R^). Cells expressing wild type FlgB or FlgC fused to Bla are ampicillin resistant (Ap^R^). Cells expressing amino acid substitutions in FlgB or FlgC that are defective in secretion are ampicillin sensitive (Ap^S^).

The bacterial flagellum can be divided into three structural components: the basal body, the hook and the filament (Fig 1) [8]. In *Salmonella*, the basal body is the flagellar motor that includes rotor, stator and rod structures. The rod acts as a drive-shaft, which transverses the periplasmic space from the cytoplasmic membrane through the cell wall to the outer membrane. The hook extends from the rod at the cell surface to a defined length of ~55 nm in *Salmonella*, which is optimal for the formation of functional flagellar bundles [9]. The hook is a flexible structure that acts as a universal joint to connect the rigid rod to the long, external filament. The filament extends ~10–20 μm from the hook-tip and depending on its rotation, clockwise or counterclockwise, will propel the bacterium in a forward direction or cause the bacterium to tumble as a mechanism to reorient the bacterium in a chemical gradient. The basal body also includes four ring structures. Flagellum assembly initiates with the formation of the MS-ring, consisting of ~34 subunits of a single protein, FliF, in the cytoplasmic membrane (Fig 1) [10–12]. Beneath the MS-ring is the cytoplasmic C-ring, which consists of FliG, FliM and FliN and acts as a bi-directional rotor [13]. The basal body also contains a bushing composed of P(FlgI)- and L(FlgH)-ring structures in the periplasm and lipopolysaccharide (outer membrane), respectively [14,15].

The majority of the flagellar structure is extracellular. Protein subunits are secreted through a T3S system, which assembles within the MS-ring (Fig 1). The core flagellar T3S apparatus of *Salmonella* consists of FliP, FliQ and FliR in the stoichiometry FliP_5_Q_4_R_1_ [16]. The FliP, FliQ and FliR proteins have predicted transmembrane segments, yet once assembled, the FliP_5_Q_4_R_1_ complex is located above the predicted inner membrane location. A single copy of FlhB directly associates with the FliP_5_Q_4_R_1_ complex. A nonamer of the FlhA protein associates with the FlhB_1_- FliP_5_Q_4_R_1_ complex [17]. Each FlhA monomer has eight predicted transmembrane segments in its N-terminus followed by a large C-terminal cytoplasmic domain where subunits to be secreted are delivered [18,19].

A unique feature of T3S systems is the ability to change secretion substrate specificity from one class of protein substrates to a completely different class of substrates [8,20]. In the flagellar T3S system early secretion substrates form the rod and hook structures of the flagellum. Following completion of the hook-basal body (HBB), the flagellar T3S apparatus switches specificity to late substrates. Late structures include the proteins that make up the filament (FliC or FljB) and hook associated proteins or HAPs. There are three HAP proteins FlgK, FlgL and FliD. FlgK and FlgL form a hook-filament junction and FliD is the filament cap, which acts as a scaffold to allow secreted FliC or FljB subunits to fold and assemble on the tip on the growing filament.

The flagellar T3S-specificity switch is part of a mechanism that controls the length of the hook structure. When the hook reaches its terminal length of ~55 nm, hook subunits are no longer secreted. A switch occurs to prevent further hook subunit secretion and allow for filament subunit secretion and polymerization onto the completed hook. The flagellar T3S-specificity switch involves the interaction of an intermittently secreted molecular ruler protein, FliK [22], with a switch protein, FlhB [23,24]. A structure of the N-terminus of FlhB (FlhB_N_) in complex with FliP_5_Q_4_R_1_ has been solved and demonstrated that a single subunit of FlhB_N_ associates with the FliP_5_Q_4_R_1_ core [25]. Another aspect of the T3S-specificity switch is the requirement that FlhB undergo autocleavage between residues N269 and P270 of the 383 amino acid protein [26]. In strains either deleted for *fliK* or defective in FlhB autocleavage, cells are unable to undergo the secretion specificity switch. This results in a polyhook phenotype as FlgE (hook subunit) secretion and polymerization continues well beyond the normal 55 nm wild-type hook-length [27,28]. One hypothesis is that once the hook reaches its terminal length, the C-terminus of FliK in the secretion channel interacts with the cleaved C-terminal domain of FlhB (FlhB_CCD_) resulting in a conformational change in FlhB_CCD_ such that early substrates are no longer recognized by the flagellar T3S apparatus [29]. Recently, photo-crosslinking experiments were used to identify the binding of the FliK C-terminal domain with the FlhB_CCD_ [23,24].

A subject of intense investigation and controversy over several decades regards the nature of the T3S signal. All T3S substrates possess an N-terminal peptide signal that lacks a defined, conserved sequence of amino acids. In the flagellar T3S system, it was shown that the N-terminal amino acids of export substrates are all disordered in their monomeric form in solution [30]. It is also known that late secretion substrates require both an N-terminal peptide signal and a T3S-chaperone, which targets late substrates for secretion [31–33]. Thus, in a current model the T3S-specificity switch transitions from a requirement of an N-terminal peptide signal for early secretion substrates to a requirement for both an N-terminal peptide signal and a T3S-chaperone for late secretion substrates. T3S-chaperones direct their cognate substrates to the large C-terminal cytoplasmic domain of FlhA that resides beneath the core T3S apparatus (Fig 1A) [34–38]. How a conformational change in the FlhB_CCD_ could result in a requirement for T3S-chaperone binding to FlhA to target late substrates remains to be determined.

The purpose of this study was to investigate the nature of early flagellar substrate secretion signals. The early flagellar secretion-substrates are required for the structure and formation of the Rod-Hook component of the HBB. The flagellar rod can be divided into proximal and distal halves. Proximal rod assembly initiates with the secretion and assembly of FliE above the FlhB_1_-FliP_5_Q_4_R_1_ core followed by FlgB, FlgC and FlgF. The FliE protein plays dual roles in the assembly of the *Salmonella* flagellum as the final component of the flagellar type III secretion system (fT3SS) and as an adaptor protein that anchors the proximal rod to the membrane imbedded MS-ring structure [39–41]. FlgJ is thought to assemble on the completed proximal rod and is only transiently associated during assembly. FlgJ caps the proximal rod and acts a scaffold for polymerization of the distal rod protein, FlgG. FlgJ is also a muramidase, which digests the cell wall above the proximal rod allowing FlgG polymerization to the outer membrane completing the rod structure [42,43].

Traditional secretion of T3S substrates is evaluated by Western blot analysis of secreted substrates. In our lab, we have developed β-lactamase lacking its Sec secretion signal (Bla) as a reporter for secretion of flagellar proteins into the periplasm through the flagellar type III secretion (T3S) system [40,41,44–47]. Bla must be secreted into the periplasm to fold into an active conformation in order to confer resistance to ampicillin (Ap^R^). The use of Bla as a reporter for flagellar T3S allows for both a positive selection for secretion and for the quantification of secreted protein levels. We chose to characterize the FlgB and FlgC early secretion signals because they are the first true rod subunits secreted and with lengths of 138 and 134 residues, respectively, they are relatively small. Evans et al. reported that purified FlhB_CCD_ binds the hook cap protein FlgD, which is also an early secretion substrate [48]. Small deletions revealed residues within a hydrophobic pocket of the FlhB_CCD_, which when deleted, no longer bound FlgD. This study revealed that residue F45 in FlgB is essential for secretion of FlgB. We tested for effects of the hydrophobic pocket of the FlhB_CCD_ on recognition of the F45 residue of FlgB for secretion and found genetic evidence for a direct interaction. Recently, Bryant et al. performed a more detailed characterization of the FlgD interaction with the FlhB_CCD_ hydrophobic patch and identified a critical F39 that helps target FlgD for secretion [49]. Those studies together with our findings with FlgB suggests some conservation in substrate recognition for early flagellar secretion substrates by the FlhB_CCD_.

## Results

### In-frame deletions in FlgB-Bla and FlgC-Bla implicate regions in FlgB and FlgC important for secretion

The purpose of this study was to identify sequences of early flagellar type III secretion (T3S) substrates that were important for export through the flagellar T3S system. *Salmonella* bacteria expressing full-length fusions of early secretion substrates FlgB, FlgC, FlgF, FlgG and FlgJ to Bla from their native, chromosomal loci were ampicillin resistant (Ap^R^) in the presence of a functional flagellar T3S apparatus indicating that the fusion proteins are secreted into the periplasm where Bla can fold into an active conformation (S1 Table). In-frame 10 amino acid deletions starting from the C-terminal amino acid codon of *flgB* or *flgC* were generated at the chromosomal *flg* locus (Methods).

**Fig 2:**
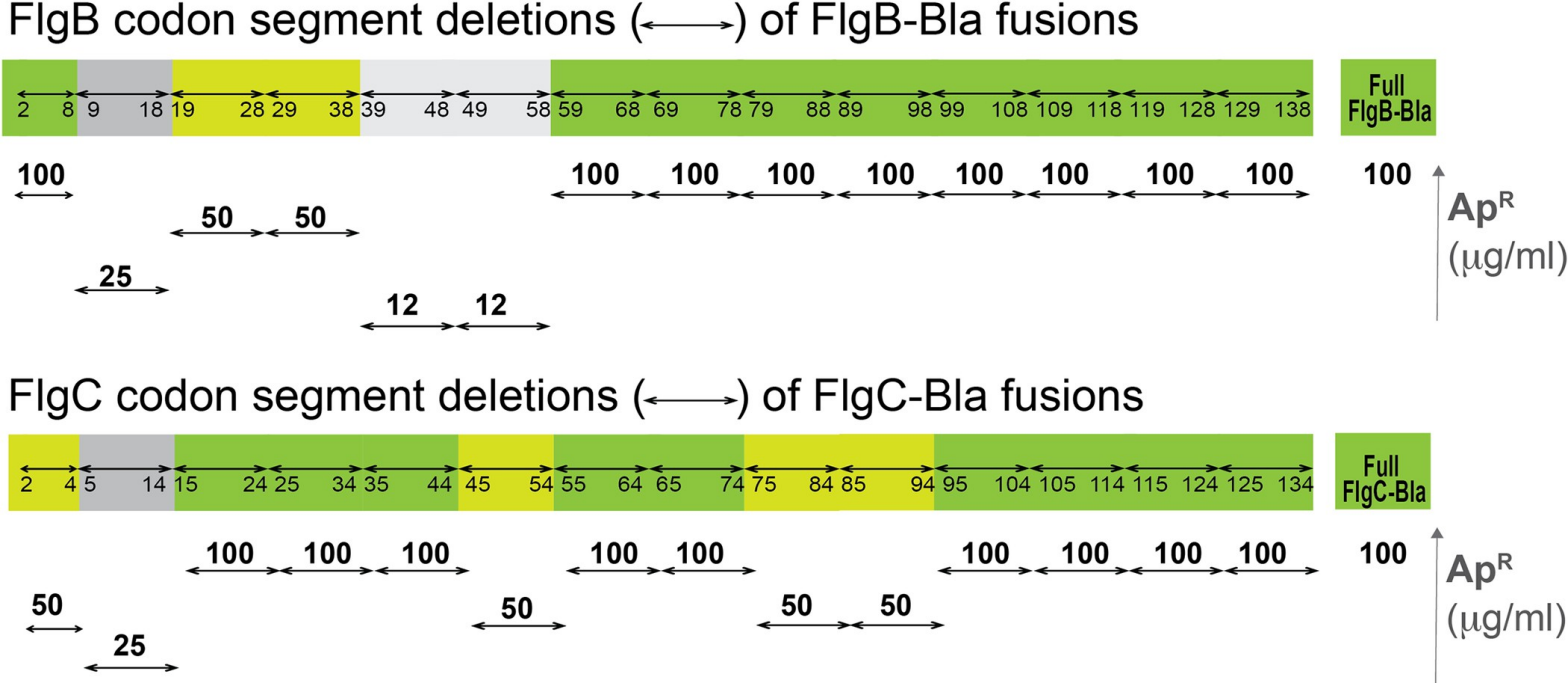
Effect of in-frame deletions in FlgB and FlgC on the secretion of FlgB-Bla and FlgC-Bla into the periplasm. Ten amino acid in frame codon deletions were constructed in *flgB* and *flgC* starting with the C-terminus. The numbers underneath the double arrow in the colored line indicate which amino acids were deleted in each gene. The MIC values of the full length FlgB-Bla or FlgC-Bla was 100 μg/ml. The MIC values of each deletion is reported using colors (green for 100 μg/ml; yellow for 50 μg/ml; dark grey for 25 μg/ml and lighter grey for 12 μg/ml). A visual representation of the secretion of each deletion is also shown underneath showing the MIC values for each deleted segment fusion above the double arrows.

For FlgB, deletion of 10 amino acid segments starting from codon 9 through codon 58 all showed reduced FlgB-Bla secretion levels (Fig 2). The most significant reduction in FlgB-Bla secreted levels was observed with deletion of either codons 39 through 48 or codons 49 through 58, which each gave an MIC value for Ap of 12 μg/ml compared to 100 μg/ml for the full length FlgB-Bla construct. Deletion of 10 amino segments after codon 58 had no measurable effect on FlgB-Bla secretion levels suggesting the C-terminal half of FlgB (codons 59 through 138) were not involved in substrate targeting of FlgB to the flagellar T3S apparatus. However, the possibility that these deletions could affect protein expression or stability had not been ruled out.

The effects of 10 amino acid deletions on secreted levels of the Bla fusions were different for FlgC as compared to FlgB. A 10 amino acid deletion of codons 5 through 14 resulted in an MIC for Ap of 25 μg/ml compared to 100 μg/ml for the full length FlgC-Bla construct. Three other 10 amino acid deletions, codons 45–54, 75–84 and 85–94 resulted in MIC levels of 50 μg/ml as did a shorter N-terminal deletion of codons 2–4 (Fig 2). These results suggest that amino acids in the very N-terminus of FlgC (residues 5 through 14) are important, for secretion, but the downstream residues were not as significant for secretion as was observed with FlgB.

### Identification of residues in FlgB affecting secretion and/or assembly of FlgB between amino acids 39 through 48

The in-frame deletion experiment presented above suggested that the region between amino acids 39 and 58 of FlgB might be critical for secretion of FlgB-Bla. This region was screened for single amino acids that were important residues for FlgB secretion and/or assembly. Because FlgB assembly is required for completion of the hook-basal body (HBB) and the subsequent secretion-specificity switch from early to late substrate secretion, we utilized a Mud-*lac* operon reporter fusion to the σ^28^-dependent class 3 *fljB* promoter (*fljB*::MudJ) [21]. MudJ is a transposable element used to fuse the *lac* operon to any promoter of interest for the characterization of the regulation of transcription of that gene and to provide classical Lac selections and screens to characterize transcriptional regulation for any gene of interest [50]. A mutant in *flgB* that is defective in HBB assembly would result in accumulation of the late secretion substrate FlgM in the cytoplasm. FlgM is an anti-σ^28^ factor that is secreted after HBB completion to couple filament gene transcription to completion of the HBB (Fig 1) [51]. A *flgB* or *flgC* mutant that is defective in HBB assembly retains FlgM in the cytoplasm, which inhibits σ^28^-dependent transcription of the *fljB*::MudJ (*fljB-lac*) reporter and results in a a Lac^-^ phenotype (Fig 1B) [51].

Amino acid codons 39 through 58 of *flgB* were mutagenized using a doped oligonucleotide (see Methods). Mutants with various levels of Lac phenotypes were purified, re-tested for their Lac phenotypes and sequenced. S2 Table lists the mutants for which single base pair mutations were obtained. Sanger sequencing of 44 individual mutants between position 39 though 48 resulted in sequences with a variety of single mutations at all amino acid positions in the doped segment, indicating the mutagenesis worked well. Of the 44 single mutants isolated between amino acid codons 39 through 48 of *flgB*, 18 mutants resulted in reduced *fljB*::MudJ expression indicating a defect in HBB assembly. Of these 18 mutants, 8 different substitutions were obtained at amino acids 41, 42, 43 and 45 and are reported in Table 1. Of note, twelve of the 18 mutants were a substitution of amino acid F45. All the *flgB* alleles reported in Table 1 exhibited reduced motility and a defect in HBB assembly as evident by a reduction in *fljB*::MudJ transcription.

**Table 1:** Flagellar class 3 expression phenotypes, motility and secretion of *flgB* alleles assembly defective.

FlgB amino acid codon	Substitution Mutation	Occurrence	Lac phenotype^a^	Motility phenotype^b^	Ampicillin resistance^c^
41	R41D	x1	+/-	10%	75
42	D42Y	x2	+/-	10%	75
43	I43S	x3	+/-	5%	100
45	F45C	x1	+/-	8%	5
45	F45I	x2	+/-	20%	5
45	F45L	x6	+/-	17%	5
45	F45M	x1	+/-	20%	5
45	F45V	x2	+/-	34%	5
control	WT		++++	100%	75

^a^All strains carried *fljB*5001::MudJ Δ*hin*-*5718*::FRT alleles to assay for σ^28^-dependent class 3 flagellar gene transcription that was determined on Mac-Lac and TTC-lac indicator medium (37°C) (++++: TTC-Lac white and ML dark red; +++: TTC-Lac pink and ML dark red; ++: TTC-Lac red and Mac-Lac red; +: TTC-Lac dark red and ML pink; +/-: TTC-Lac dark red and Mac-Lac light pink;—: TTC-Lac dark red Mac-Lac white)

^b^Motility phenotypes are given as percentage of WT motility at 37°C

^c^*flgB* alleles were moved into a *flgB-bla* fusion expressed at the chromosomal *flg* locus. Ampicillin resistance levels were assayed on PPBS-Ap plates with varying ampicillin concentrations.

In order to separate amino acid substitutions in FlgB that were defective in secretion from those defective in assembly, the substitutions were introduced into a *flgB-bla* reporter fusion expressed at the chromosomal *flgB* locus. An Ap^R^ phenotype is indicative that the FlgB-Bla fusion is expressed and secreted into the periplasm (Fig 1C). Thus, we are able to screen *flgB* alleles that are assembly-defective but secretion-competent from those that are secretion-defective. An amino acid substitution in FlgB that was specific to FlgB assembly and not secretion was expected to have a high level of Ap^R^ similar to the wild-type FlgB-bla construct while a substitution defective in secretion was expected to exhibit reduced Ap^R^ levels. Of the 8 *flgB* alleles reported in Table 1, all the substitutions for amino acid F45 showed a severe secretion defect implicating residue F45 of FlgB as critical for secretion while substitutions R41D, D42Y and I43S exhibited Ap^R^ levels close to the wild-type (Table 1). We conclude that the R41D, D42Y and I43S are defective in assembly.

The doped oligonucleotide mutagenesis between amino acids 49 through 58 primarily yielded mutants containing multiple mutations that were discarded, and only a few single mutations at some of the positions in the doped region were obtained (S2 Table). These mutants were not followed further as we decided to focus on residue F45 of FlgB for the rest of this study.

### Residue F45 is important for FlgB secretion

Since the 5 amino acid substitutions at F45 (F45C, F45I, F45L, F45M, and F45V) in FlgB found through the doped oligo mutagenesis experiment described above were defective in secretion (Table 1), codon 45 in *flgB* was randomized using an oligonucleotide with all possible DNA sequence combinations (NNN) at codon 45. Alleles coding for all twenty amino acids of *flgB* codon 45 were obtained and tested for effects on HBB assembly as described above (Fig 1B). All the amino acid substitutions, but the wild type (F45) and Y45, affected normal HBB assembly (Table 2).

**Table 2:** Flagellar class 3 expression phenotypes and motility of *flgB* and *flgC* alleles expressed from their native locus and mutagenized at F45 or F49, respectively.

	FlgB (aa45)		FlgC (aa49)	
Amino Acid	^a^Lac	^b^Motility	^a^Lac	^b^Motility
ALA	-	5%	++	75%
VAL	+	20–40%	++++	101%
ILE	++	42%	++++	94%
LEU	++	40%	++++	100%
MET	+	31%	+++	88%
**PHE**	**++++**	**100%**	**++++**	**100%**
TYR	++++	100%	++++	97%
TRP	+++	60%	+++	83%
HIS	+	14%	++	40%
LYS	-	0%	-	0%
ARG	-	0%	-	0%
ASP	-	0%	-	0%
GLU	-	5%	+++	88%
ASN	-	0–5%	++	44%
GLN	-	0%	-	0%
SER	-	5%	++	47%
THR	+	14–19%	++++	110%
CYS	+	20–22%	+++	69%
GLY	-	0%	-	0%
PRO	-/+	5%	-/+	0%

^a^Strains carried Δ*flgBC fljB*5001::MudJ Δ*hin*-*5718*::FRT alleles to assay for σ^28^-dependent class 3 flagellar gene transcription which was determined on Mac-Lac and TTC-Lac indicator medium (37°C) (++++: TTC-Lac white and ML dark red; +++: TTC-Lac pink and ML dark red; ++: TTC-Lac red and Mac-Lac red; +: TTC-Lac dark red and ML pink; +/-: TTC-Lac dark red and Mac-Lac light pink;—: TTC-Lac dark red Mac-Lac white)

^b^Motility phenotypes are given as percentage of WT motility at 37°C.

The *flgB* alleles with codon 45 substitutions were also placed in front of β-lactamase, this time at the *araBAD* locus under control of the arabinose-inducible *araBAD* promoter (P_*araBAD*_). This allowed us to test for the ability of different FlgB-Bla codon 45 substitutions to compete with the wild type FlgB for secretion and inhibition of HBB assembly. We have previously shown that the presence of bile salts increases sensitivity to Ap on solid media, which allows for the detection of small differences in levels of Ap^R^ [44]. On proteose peptone bile salts (PPBS) solid medium (see Methods), we observed significant secretion for three of twenty FlgB-Bla codons: F45, W45 and Y45 (Table 3). However, the Y45 and W45 showed reduced Ap^R^ compared to F45. The Y45 and W45 substitutions grew similar to F45 on solid medium containing arabinose (PPBS-Ara) and 5 μg/ml Ap, but exhibited Ap-sensitivity at higher Ap concentrations that did not affect the F45 allele tested up to 15 μg/ml. All other substitutions did not grow at the lowest level of Ap (5μg/ml) tested. These results suggest that full wild-type FlgB secretion occurs only with phenylalanine at codon 45 of FlgB and all other amino acid substitutions are defective. We also noted that expression of wild-type (F45) FlgB-Bla from P_*araBAD*_ had an inhibitory effect on normal HBB assembly resulting in the inhibition of σ^28^-dependent transcription of the *fljB*::MudJ reporter. No other amino acid substitution showed this inhibitory effect (Table 3). We conclude that FlgB-Bla competes with FlgB for secretion and assembly into the growing basal body. The incorporation of a FlgB-Bla fusion into the assembling basal body appears to inhibit further rod assembly.

**Table 3:** Secretion phenotypes of FlgB-Bla fusions expressed from P_*araBAD*_ with amino acid substitutions at codon 45 of FlgB. The strains carried the *fljB5001*::MudJ Δ*hin-5718*::FRT alleles to determine the effect of *flgB-bla* secretion on HBB assembly. Inhibition of HBB assembly would result in accumulation of FlgM in the cytoplasm and inhibition of σ^28^-dependent transcription of the *fljB*::MudJ reporter.

Strain number^a^	FlgB F45nnn^a^	PPBS-Ara-Ap^b^				Lac^c^	Ara-Lac ^c^	Expression ^d^
		5	7.5	10	15			
TH27277	A	-	-	-	-	++++	++++	+++
TH27278	V	-	-	-	-	++++	++++	ND
TH27279	I	-	-	-	-	++++	++++	+++
TH27280	L	-	-	-	-	++++	++++	ND
TH27281	M	-	-	-	-	++++	++++	ND
TH27282	F (WT)	+	+	+	+	++++	-	+++
TH27283	Y	+	+/-	-	-	++++	++++	+++
TH27398	W	+	+/-	-	-	++++	++++	ND
TH27284	H	-	-	-	-	++++	++++	+++
TH27399	K	-	-	-	-	++++	++++	ND
TH27285	R	-	-	-	-	++++	++++	+++
TH27286	D	-	-	-	-	++++	++++	+++
TH27287	E	-	-	-	-	++++	++++	+++
TH27400	N	-	-	-	-	++++	++++	ND
TH27288	Q	-	-	-	-	++++	++++	+++
TH27289	S	-	-	-	-	++++	++++	+++
TH27401	T	-	-	-	-	++++	++++	ND
TH27290	C	-	-	-	-	++++	++++	ND
TH27291	G	-	-	-	-	++++	++++	+++
TH27292	P	-	-	-	-	++++	++++	+++
TH27299	Stop	-	-	-	-	++++	++++	-

^a^All strains had the following genotype: Δ*araBAD*::(*flgB*-5’UTR)-*flgB-bla* (a.k.a. P_*araBAD*_-*flgB-bla*) *fljB5001*::MudJ Δ*hin-5718*::FRT, where amino acid substitutions at codon 45 of the P_*araBAD*_-*flgB*-*bla* constructs are indicated.

^b^Ampicillin resistance was tested on PPBS-arabinose plates with varying ampicillin concentrations as shown in each column in μg/ml Ap. (+: grew; -: did not grow) None of the constructs grew on plates containing Ap5 without arabinose.

^c^Flagellar class 3 σ^28^-dependent transcription of a *fljB*::MudJ reporter fusion construct (*fljB5001*::MudJ Δ*hin-5718*::FRT) was determined on Mac-Lac and TTC-Lac indicator medium with and without added arabinose at 37°C. (++++: TTC-Lac white and ML dark red; +++: TTC-Lac pink and ML dark red; ++: TTC-Lac red and Mac-Lac red; +: TTC-Lac dark red and ML pink; +/-: TTC-Lac dark red and Mac-Lac light pink;—: TTC-Lac dark red Mac-Lac white)

^d^FlgB-Bla expression levels were determined on cell extracts by SDS-PAGE followed by western blot analyses using anti-β-lactamase antibodies. “+++” indicates well expressed; “-” indicates not expressed; ND: Not Determined.

We also performed MIC assays in liquid media lacking bile salts (LB+Ara) in order to quantify the effects of codon 45 substitutions on FlgB-Bla secretion under less stringent Ap^R^ selection conditions (Tables 4 and S3). The lack of bile salts results in relaxed selection for Ap^R^ such that one can detect differences in secretion for codon 45 substitutions that are not observed on the bile salts-containing solid medium. These assays were also done in strains deleted for the chromosomal *flgB* and *flgC* genes to ensure that all flagellar secreting structures were identical and the lack of rod structures would allow for maximal levels of secreted FlgB-Bla. Otherwise, secretion would only occur at each basal structure for the short time period after completion of the T3S apparatus and prior to rod completion. After rod completion, the Bla fusion is secreted into the spent growth medium and would not contribute to Ap^R^. Thus, Δ*flgBC* strains that are unable to assemble rod structures provides a more controlled assay for the effect of codon 45 substitutions on FlgB-Bla secretion (Fig 1C). This also allowed for the quantification of FlgB-Bla secretion independent of potential effects of the different FlgB-Bla codon 45 alleles on rod formation. The effects of codon 45 substitutions on FlgB-Bla secretion were determined by measuring MIC levels to Ap for cells grown in the presence of arabinose inducer. The results are presented in Table 4. The MIC to Ap for FlgB-Bla expressed from P_*araBAD*_ in a strain deleted for *flgBC*, was 25 μg/ml compared to 100 μg/ml for FlgB-Bla expressed from the chromosomal *flgB* locus (S1 Table). Only a conservative F45Y substitution resulted in a wild-type motility phenotype (Table 2) and an Ap^R^ MIC level similar to the wild-type F45 allele (Table 4). Polar amino acid residues exhibited intermediate levels of motility (Table 2) and Ap^R^ MIC levels (Table 4) while nonpolar, charged residues, alanine and glycine consistently exhibited substantial defects in FlgB secretion.

**Table 4:** The effect of amino acid substitutions at codon F45 of *flgB* and F49 of *flgC* on the secretion of FlgB-Bla and FlgC-Bla, in a proximal rod mutant background (Δ*flgBC*).

	^a^MIC values (μg/ml) *flgB-bla* or *flgC-bla* expressed from the P_*araBAD*_ in a Δ*flgBC* background					
Amino Acid	FlgB F45			FlgC F49		
	Assay1	Assay2	Assay3	Assay1	Assay2	Assay3
ALA	12	12	12	3	6	3
VAL	3	6	6	12	12	12
ILE	12	12	12	6	12	12
LEU	6	6	6	12	12	12
MET	3	6	6	6	6	6
PHE	25	25	25	12	12	25
TYR	25	25	25	12	12	12
TRP	12	12	12	12	12	12
HIS	12	12	12	3	3	6
LYS	6	6	6	12	12	6
ARG	6	6	6	12	12	12
ASP	3	6	6	12	12	12
GLU	6	6	6	6	6	12
ASN	6	12	12	6	12	12
GLN	6	6	6	25	25	25
SER	12	12	12	12	12	12
THR	6	6	6	12	12	12
CYS	6	6	6	12	12	12
GLY	12	12	12	12	12	12
PRO	12	12	25	12	12	25

^a^The MIC to Ap were measured in strains expressing *flgB-bla* with amino acid substitutions at codon 45 of *flgB* or *flgC-bla* with amino acid substitutions at codon 49 of *flgC* from the *araBAD* locus (P_*araBAD*_-*flgB-bla* or P_*araBAD*_-*flgC-bla)* in strains deleted for the proximal rod components (Δ*flgBC* background). MIC assays were conducted on strains grown in the presence of arabinose. No addition of arabinose resulted in MIC’s of less than 1.5 μg/ml

The Ap^R^ assays on solid medium containing bile salts provided a stringent screen that allowed us to determine that the F45Y and F45W substitutions were defective relative to the wild type F45 codon. The MIC assays in the absence of bile salts provided less stringent Ap^R^ selection, which allowed us to observe a gradient of effects of codon 45 substitutions on secretion: in general aromatic amino acids (F,Y,W) were secreted to higher levels than the remaining hydrophobic residues (V,I,L,M,C), which were secreted to higher levels than polar residues (H,S,T,N,Q), which, except for N and Q, were secreted to higher levels than small residues, proline and charged residues (A,G,P,L,R,D,E).

### Residue F49 is important for FlgC assembly

Evans et al. (2013) had reported that a conserved phenylalanine residue, present in the N-terminus of all rod and hook substrates, might be critical for secretion of early substrates [48]. For FlgB, this residue would correspond to the phenylalanine at amino acid 45, and indeed, as shown above, the effect of FlgB F45 on FlgB secretion is very clear. For FlgC, F49 was predicted to be critical for secretion [48]. However, a deletion of codons 45 through 54 of *flgC* resulted in only a slight, 2-fold reduction for MIC to Ap for a FlgC-Bla fusion (Fig 2). Alleles with the twenty amino acids at codon 49 of *flgC* were obtained and tested for hook basal body (HBB) assembly as described earlier (Fig 1B). As for FlgB, residues K, R, D, Q and G in place of F49 in FlgC prevented FlgC assembly (Table 2). Residues V, I, L, F and Y at amino acid 49 of FlgC assembled as wild type. The rest of the substitutions showed some decrease in assembly, as seen from the Lac and motility phenotypes.

FlgC F49 alleles were then placed in a *flgC-bla* construct expressed from the P_*araBAD*_ promoter in a strain deleted for *flgBC*, and their effects on FlgC-Bla secretion were determined by measuring MIC levels to Ap for cells grown in the presence of arabinose inducer (Table 4). A difficulty with this set of strains was that the MIC obtained for *flgC-bla* expressed from P_*araBAD*_ in a strain deleted for *flgBC*, was relatively low, 12 μg/ml, giving little room for detecting significant effects of amino acid substitutions on secretion. FlgC substitutions F49A, F49M, F49H and F49E resulted in reduced MIC levels for Ap at ~3, 6, ~3 and ~6 μg/ml, respectively. The F49 substitutions that exhibited a non-motile phenotype (0% motility in Table 2) were all secreted at or near wild-type levels when tested in the FlgC-Bla assay. This indicated that they were defective in assembly rather than secretion. One FlgC substitution, F49Q, resulted in higher MIC for secretion of FlgC-Bla (Table 4), but the same substitution at the chromosomal *flgC* locus resulted in a non-motile phenotype indicating a strong defect in assembly (Table 2). F49D was also well secreted (MIC = 12μg/ml), but nonmotile suggesting a defect in assembly. We note that F49D was assembly-defective while F49E showed almost wild-type motility (88%) despite the similarity between these amino acid residues.

The set of F49 substitutions in the P_*araBAD*_*-flgC-bla* constructs were placed into a strain containing the wild type chromosomal rod components and the class 3 *fljB*::MudJ reporter. Secretion of FlgC-Bla alleles were tested on PPBS solid medium containing arabinose (Table 5). Most results agreed with the MIC values performed in LB media in the proximal rod mutant background (shown in Table 4) and it is clear that FlgC F49 appeared more tolerant to amino acid substitution than FlgB F45 (compare Tables 3 and 5). Alanine and histidine were the only substitutions at amino acid 49 of FlgC-Bla that did not grow on PPBS-Ara with 5ug/ml of Ap. This suggests a defect in secretion, yet when substituted in the chromosomal *flgC* locus (no fusion) they exhibited 75% of wild-type motility for F49A and 40% for F49H (Table 2). The substitutions did not affect expression levels (S4 Fig). We hypothesize that even though they are defective in secretion, there is enough low-level secretion to assemble the 6 FlgC subunits needed to form a functional rod, but not enough is secreted in the P_*araBAD*_*-flgC-bla* constructs to confer Ap^R^ on the stringent PPBS-Ap^R^ plates.

**Table 5:** Secretion phenotypes of FlgC-Bla fusions expressed from P_*araBAD*_ with amino acid substitutions at codon 49 of *flgC*. The strains carried the *fljB5001*::MudJ Δ*hin-5718*::FRT alleles to determine the effect of FlgC-Bla secretion on HBB assembly. Inhibition of HBB assembly would result in accumulation of FlgM in the cytoplasm and inhibition of σ^28^-dependent transcription of the *fljB*::MudJ reporter.

Strain number^a^	FlgC F49nnn^a^	PPBS-Ara-Ap^b^				Lac^c^	Ara-Lac ^c^	Expression ^d^
		5	7.5	10	15			
TH27591	A	-	-	-	-	++++	++++	+++
TH27592	V	+	+	+	+	++++	-	+++
TH27593	I	+	+	+	+	++++	-	+++
TH27594	L	+	+	+	+	++++	-	+++
TH27595	M	+	+	+	+	++++	-	+++
TH27546	F (WT)	+	+	+	+	++++	-	+++
TH27596	Y	+	+	+	+	++++	+	+++
TH27597	W	+	+	+	+	++++	+++	+++
TH27598	H	-	-	-	-	++++	++++	+++
TH27599	K	+	+	+	+	++++	++++	ND
TH27600	R	+	+	+	+	++++	++++	ND
TH27601	D	+	+	+	+	++++	++++	ND
TH27602	E	+	+	+	+	++++	++++	ND
TH27603	N	+	+	+	+	++++	++++	ND
TH27604	Q	+	+	+	+	++++	++++	ND
TH27605	S	+	+	+	+	++++	++++	ND
TH27606	T	+	+	+	+	++++	+++	ND
TH27607	C	+	+	+	+	++++	+	ND
TH27608	G	+	+	+	+	++++	+++	ND
TH27609	P	+	+	+	+	++++	++++	ND

^a^All strains had the following genotype: Δ*araBAD*::*flgC-bla fljB5001*::MudJ Δ*hin-5718*::FRT, where amino acid substitutions at codon 49 of the P_*araBAD*_-*flgC*-*bla* constructs are indicated.

^b^Ap^R^ was tested on PPBS-arabinose plates with varying Ap concentrations as shown in μg/ml Ap (+: grew; -: did not grow). None of the constructs grew on plates containing Ap5 without arabinose.

^c^Flagellar class 3 σ^28^-dependent transcription of a *fljB*::MudJ reporter fusion construct (*fljB5001*::MudJ Δ*hin-5718*::FRT) was determined on Mac-Lac and TTC-Lac indicator medium with and without added arabinose at 37°C. (++++: TTC-Lac white and ML dark red; +++: TTC-Lac pink and ML dark red; ++: TTC-Lac red and Mac-Lac red; +: TTC-Lac dark red and ML pink; +/-: TTC-Lac dark red and Mac-Lac light pink;—: TTC-Lac dark red and Mac-Lac white).

^d^FlgC-Bla levels were determined on cell extracts by SDS-PAGE followed by western blot analyses using anti-β-lactamase antibodies. “+++” indicates well expressed; “-” indicates not expressed; ND: Not Determined.

Just as for FlgB-Bla, we noted that expression of FlgC-Bla from P_*araBAD*_ had an inhibitory effect on normal HBB assembly resulting in the inhibition of σ^28^-dependent transcription of the *fljB*::MudJ reporter. Substitutions of amino acid 49 of FlgC with V, I, L and M also showed this inhibitory effect (Table 5). C, Y, W, T, and G had also some level of inhibitory effects, but none of the other substitutions exhibited this effect when secreted. Residues such as K, R, D and Q seemed to allow FlgC secretion but not assembly (see Tables 2 and 5). It appears that only some alleles of FlgC-Bla can assemble into the growing basal body to inhibit further rod assembly. We conclude that the F49 position in FlgC is not as critical for FlgC secretion as is F45 of FlgB, but there is selectivity for residues required for assembly.

### The C-terminus of FlhB is a target for FlgB-Bla secretion

The flagellar type 3 secretion (T3S) system undergoes a secretion-specificity switch from early rod-hook secretion substrates to late secretion substrates, which include filament subunits and the anti-σ^28^ factor FlgM. The secreted molecular ruler protein FliK determines hook-length completion and catalyzes the secretion-specificity switch at the FlhB component of the flagellar T3S system [22,52]. The T3S-specificity switch also requires that FlhB undergo a spontaneous autocleavage event between residues N269 and P270 of the 383 amino acid protein [26]. The cleaved N-terminal domain of FlhB associates with FliP_5_Q_4_R export gate while the location of the cleaved C-terminal domain of FlhB (FlhB_CCD_) within the T3S apparatus is not known [25]. A null mutant in *fliK* or a mutation in *flhB* that is unable to undergo autocleavage are unable to switch, which results in a polyhook phenotype [26–28]. The FlhB_CCD_ structure includes a hydrophobic pocket of four residues, A286, P287, A341 and L344, [48]. Substitution of individual residues to glutamate prevented secretion of either distal rod protein FlgG or the FlgE hook protein subunits. This hydrophobic pocket was proposed to be a docking site for early secretion substrates through interaction with a critical phenylalanine residue present in the N-terminus of early secretion substrates. Deletion of F36 through L40 of FlgD prevented secretion of FlgD subunits [48]. More recently, residues 2 through 5 and 36 through 40 of FlgD, were identified as critical for secretion [49]. This included the phenylalanine residue at codon 36. Our results suggest that F49 of FlgC is not critical for FlgC secretion, but F45 of FlgB is.

In order to test whether the hydrophobic pocket of the FlhB_CCD_ was important in recognition of F45 for secretion of FlgB, codons 286, 341 and 344 of the hydrophobic pocket were targeted for VNN codon mutagenesis simultaneously (see Methods) and screened for mutations able to suppress *flgB* secretion-defective alleles at codon 45. The proline residue 287 of FlhB was not mutated since it could impact the overall structure of the FlhB_CCD_. The targeted mutation of codons 286, 341 and 344 of *flhB* as described would result in 110,592 different codon combinations. The wild-type codon sequence is predicted to occur in one out of ~2,000 mutants. Approximately 150,000 *flhB* 286VNN 341VNN 344VNN mutants were generated (see Methods).

**Fig 3:**
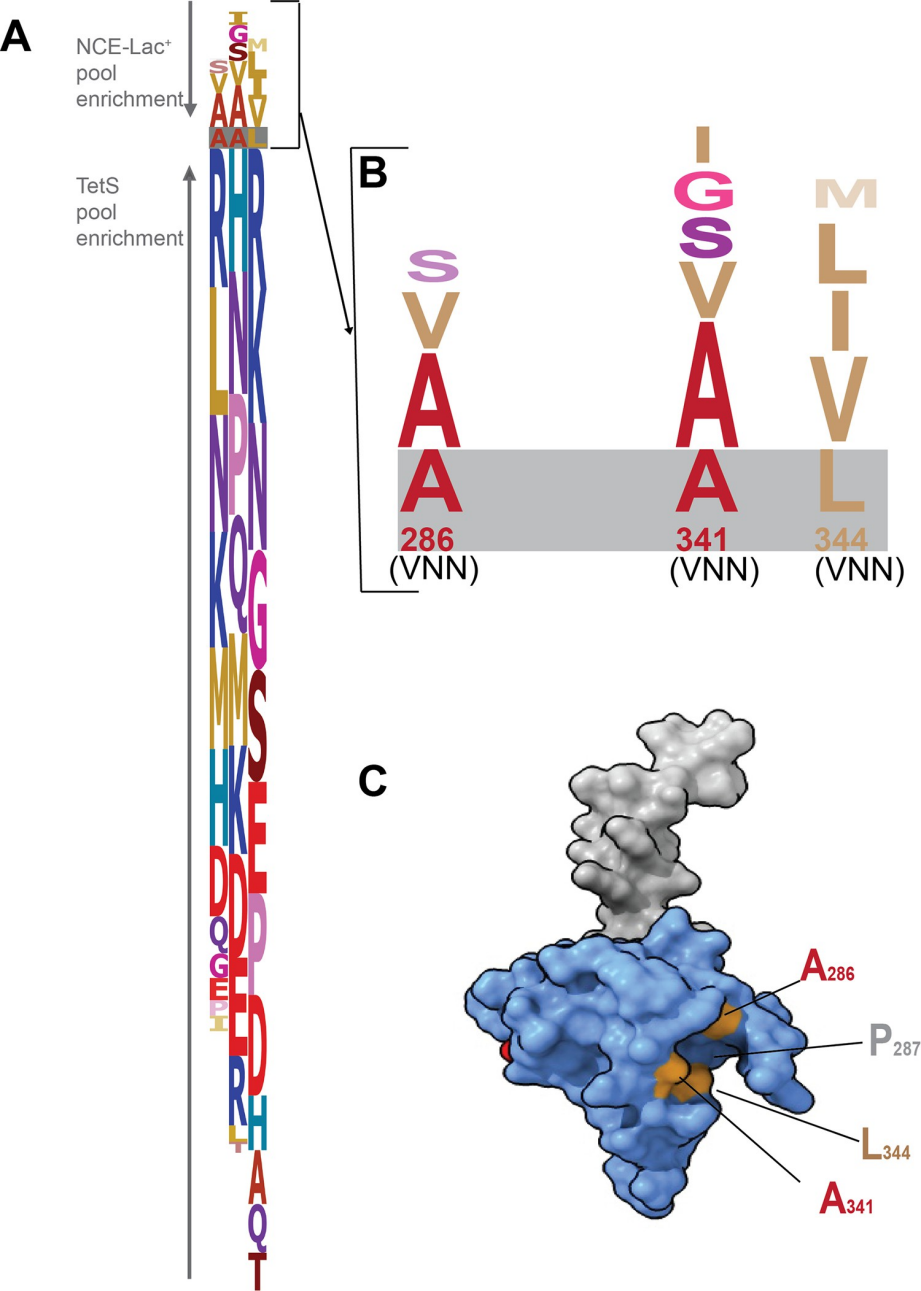
Mutagenesis of the FlhB C-terminus surface exposed hydrophobic pocket (FlhB A286VNN A341VNN L344VNN) in a *fljB5001*::MudJ Δ*hin-5718*::FRT background to determine the effect of FlhB hydrophobic pocket substitutions on HBB assembly. Approximately 1,000 mutants from the Tc^S^ pool and from the Lac^+^ pool were analyzed using next generation sequencing. The diagram represents the log odds score calculated as (log2(Lac^+^ count /Tc^S^ count)) for each amino acid at each position. The Lac^+^ (NCE-lac pool) mutants represent the mutants forming a functional HBB structure (Panel A-top and enlarged in Panel B). Panel C shows the positions of the residues (A286, A341 and L344) mutated in the FlhB C-terminus surface exposed hydrophobic pocket (PDB 3B0Z; [53]).

Preliminary experiments (See Methods) showed that the residues in the surface-exposed hydrophobic pocket in the FlhB_CCD_ were not under a strict selection for FlhB function. It appeared that the ideal FlhB residues for optimal secretion and assembly of hook basal body are A, V or S for residue 286, A, V, S, G or I for residue 341 and L, I or V for residue 344 (Fig 3). A plasmid vector expressing *flhB* with a L344A substitution complemented an *flhB* null mutant [38]. Alanine at residue 344 of FlhB was not enriched in our NCE-lac pool (selecting for HBB completion) compared to the Tc^S^ pool of total mutants (the 150,000 *flhB* mutant combination). Our method suggests that residues such as leucine, isoleucine and valine work best for FlhB function, when *flhB* is expressed from its native chromosomal locus.

**Fig 4:**
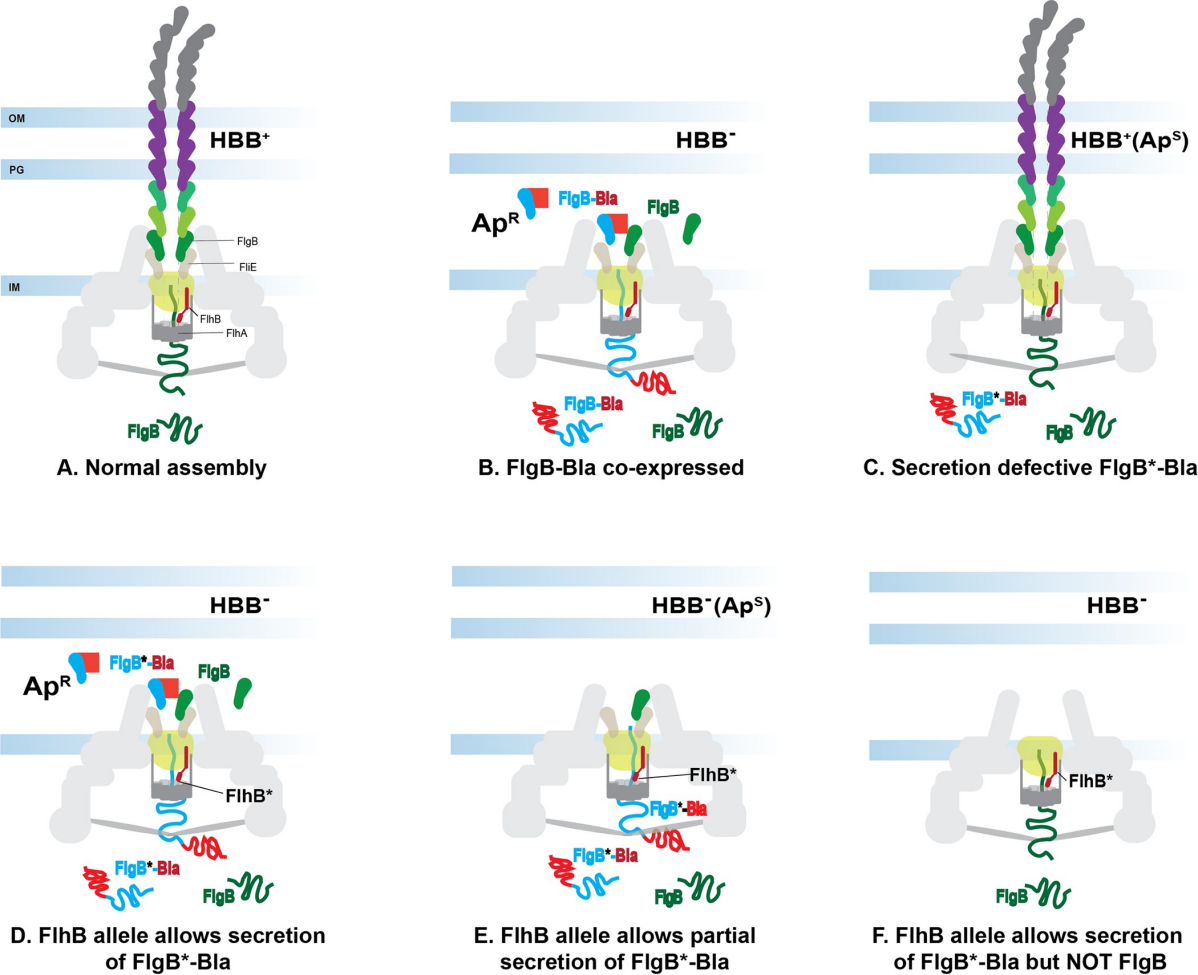
Illustration showing the secretion of FlgB and FlgB-Bla alleles in different genetic backgrounds. Panel A represents normal secretion/assembly of FlgB. When FlgB-Bla is co-expressed, we propose that FlgB-Bla assembles into the structure and interferes with further rod assembly that prevents FlgM secretion and results in a Lac^-^ phenotype (Panel B). A secretion defective FlgB*-Bla allele is not secreted and therefore does not interfere with rod assembly (Panel C). Panels D, E and F include mutants in *flhB* (FlhB*) that allow FlgB*-Bla alleles to be secreted, but constitute two classes: FlgB*-Bla alleles that are fully secreted (Panel D and F) or those stuck in the secretion apparatus (Panel E). Alleles of FlhB were also obtained that secrete mutant, FlgB*-Bla, but lose the ability to secrete wild type FlgB (Panel F).

Our goal was to use the pool of targeted FlhB_CCD_ substitutions (150,000 *flhB* 286VNN 341VNN 344VNN mutants pool) to screen for suppressors that permitted the secretion of FlgB-Bla fusions with substitutions for amino acid F45. These F45 FlgB-Bla mutants are defective in secretion by a wild-type FlhB-containing flagellar T3S system. If the hydrophobic pocket in the FlhB_CCD_ was a target for recognition of FlgB through the F45 residue, it might be possible to alter the hydrophobic pocket to recognize amino acid substitutions at position 45 and allow for mutant FlgB-Bla secretion. Twenty constructs, each with a different amino acid substitution at codon 45 of *flgB-bla*, were expressed at the *araBAD* (P_*araBAD*_-*flgB-bla*) locus leaving the chromosomal *flgB* gene intact and harboring the *fljB*::MudJ reporter to assay defects in HBB assembly. The parent strain, P_*araBAD*_-*flgB-bla fljB*::MudJ is Lac^+^ (ie. HBB^+^) in the absence of arabinose inducer (Fig 4A) and Lac^-^ (HBB^-^) when FlgB-Bla is expressed by addition of arabinose (Fig 4B). The parent strain also grows on 30 μg/ml Ap plates with added arabinose (see TH27282 in Table 6 and Fig 4B).

**Table 6:** Characterization of alleles in *flhB* that allowed secretion of FlgB-Bla fusions expressed from P_*araBAD*_ with amino acid substitutions at codon 45 of *flgB*. The strains also carried the *fljB5001*::MudJ Δ*hin-5718*::FRT alleles to determine the effect of FlgB-Bla secretion on HBB assembly. Inhibition of HBB assembly would result in accumulation of FlgM in the cytoplasm and inhibition of σ^28^-dependent transcription of the *fljB*::MudJ reporter.

Strain^a^	FlgB^b^	Lac^c^		PPBS-Ara-Ap^d^						FlhB sequence^e^		
		no ara	ara	5	7.5	10	15	30	50	A286 (gcg)	A341 (gcg)	L344 (ttg)
TH27282	F45	++++	-	+	+	+	+	+	-	*wild type flhB*		
TH27283	F45Y	++++	++++	+	+/-	-	-	-	-	*wild type flhB*		
TH27519	F45Y	+++	-	+	+	+	+	+	-	A (gct)	A (gca)	Q(caa)
TH27520	F45Y	++	-	+	+	+	+	-	-	V (gtc)	A (gcc)	Q(caa)
TH27521	F45Y	+++	-	+	+	+	+	-	-	V (gtc)	A (gct)	H (cat)
TH27522	F45Y	++++	++	+	+	+	+	+	-	V (gtc)	A (gca)	V (gta)
TH27523	F45Y	-	-	+	+	+	+	-	-	P (ccg)	A (gct)	H (cat)
TH27398	F45W	++++	++++	+	+/-	-	-	-	-	*wild type flhB*		
TH27524	F45W	++++	++/-	+	+	+	+	+	-	I (att)	A (gca)	V (gtt)
TH27525	F45W	++++	++	+	+	+	+	+	-	Y (tac)	A (gca)	V (gta)
TH27526	F45W	++++	++	+	+	+	+	+	-	V (gta)	A (gca)	V (gta)
TH27527	F45W	++++	++/-	+	+	+	+	+	-	P (cct)	A (gca)	I (ata)
TH27280	F45L	++++	++++	-	-	-	-	-	-	*wild type flhB*		
TH27528	F45L	++++	+	-	-	-	-	-	-	P (cca)	V (gtg)	L (ctc)
TH27529	F45L	+++	-	-	-	-	-	-	-	V (gtg)	T (acg)	L (cta)
TH27530	F45L	++	-	-	-	-	-	-	-	T (aca)	T (acc)	M (atg)
TH27277	F45A	++++	++++	-	-	-	-	-	-	*wild type flhB*		
TH27531	F45A	++	-	+	+/-	-	-	-	-	A (gcg)	R (agg)	L (cta)
TH27532	F45A	+	-	+	+/-	-	-	-	-	Q (cag)	I (atc)	L (ctt)
TH27289	F45S	++++	++++	-	-	-	-	-	-	*wild type flhB*		
TH27533	F45S	+	-	+	-	-	-	-	-	A (gcc)	G (ggg)	A (gca)
TH27534	F45S	+++	-	+/-	-	-	-	-	-	T (acg)	E (gag)	L (cta)
TH27277	F45T	++++	++++	-	-	-	-	-	-	*wild type flhB*		
TH27535	F45T	+++	-	+	+/-	-	-	-	-	N (aac)	A (gcg)	M (atg)
TH27290	F45C	++++	++++	-	-	-	-	-	-	*wild type flhB*		
TH27536	F45C	++	-	+	+/-	-	-	-	-	G (ggc)	T (aca)	I (ata)
TH27537	F45C	++	-	+	+/-	-	-	-	-	A (gct)	R (agg)	L (ctt)
TH27285	F45R	++++	++++	-	-	-	-	-	-	*wild type flhB*		
TH27538	F45R	++	-	+	+/-	-	-	-	-	A (gca)	V(gtc)	E (gag)

^a^All strains had the following genotype: Δ*araBAD*::(*flgB*-5’UTR)-*flgB-bla* (P_*araBAD*_-*flgB*-*bla*) *fljB5001*::MudJ Δ*hin-5718*::FRT and FlhB A286NNN A341VNN L344VNN, where amino acid substitutions at codon 45 of the P_*araBAD*_-*flgB*-*bla* constructs are indicated and the *flhB* mutation reported. The detailed genotype is listed on S6 Table.

^b^Indication of the amino acid substitution at position 45 of FlgB expressed from P_*araBAD*_-*flgB*-*bla*

^c^Flagellar class 3 σ^28^-dependent transcription of a *fljB*::MudJ reporter fusion construct (*fljB5001*::MudJ Δ*hin-5718*::FRT) was determined on Mac-Lac and TTC-lac indicator medium with and without added arabinose (++++: TTC-Lac white and ML dark red; +++: TTC-Lac pink and ML dark red; ++: TTC-Lac red and Mac-Lac red; +: TTC-Lac dark red and ML pink; —: Mac-Lac white)

^d^Ampicillin resistance was tested on PPBS- plates with and without arabinose (PPBS-Ap5) and with varying ampicillin concentrations as shown in μg/ml Ap. None of the strains grew on PPBS-Ap5 without arabinose. (+: grew well; +/-: grew a little; -: did not grow)

^e^*flhB* mutations, as obtained by DNA sequence analysis

The twenty strains, containing a different amino acid substitution at codon 45 of *flgB-bla*, carried a *tetRA* cassette insertion replacing codons 286 through 344 of *flhB*. The Δ*flhB*::*tetRA* cassette was replaced by P22-mediated transduction with phage grown on the pool of targeted FlhB_CCD_ substitutions followed by selection on Tc^S^ selective media. A dilution of P22 transducing lysate was used that gave ~5,000 Tc^S^ transductants per plate. The ~5,000 Tc^S^ transductants obtained were then pooled, grown in LB with arabinose and plated onto PPBS-Ap5-Ara plates to screen for *flhB* mutants that would secrete the different FlgB-Bla constructs. For seventeen constructs the number of Ara-Ap^R^ recombinants was low (S5 Table). Substitutions F45V, F45I, F45M, F45K, F45Q, and F45P as recipients yielded no Ara-Ap^R^ recombinants. Eleven recipients yielded few Ara-Ap^R^ recombinants: F45A(5), F45L(1), F45H(3), F45R(16), F45D(4), F45E(2), F45N(1), F45S(7), F45T(1), F45C(2) and F45G(1). The wild-type F45(>2,000) and aromatically related substitutions F45Y(>500) and F45W(>200) yield significantly higher numbers of Ara-Ap^R^ recombinants than the other seventeen recipients.

Twenty Ara-Ap^R^ recombinants were marker-rescued and tested on Lac indicator media in the presence and absence of Ara to determine if the mutated *flhB* gene could produce a functional flagellar T3S apparatus (Lac^+^ without added Ara, Fig 4A), or if expression of the FlgB-Bla fusion could prevent the assembly of a functional flagellar T3SS (Lac^-^ with added Ara inducer, Fig 4B). The results are shown in Table 6. All *flhB* alleles that showed wild type levels of *fljB*::MudJ expression had either a leucine or valine codon at position 344 of *flhB*. This is consistent with the preliminary experiments showing that leucine, isoleucine and valine at amino acid 344 of FlhB worked best for FlhB function. All the mutants tested showed a defect in flagellar T3S assembly in the presence of Ara. The levels of Ara-Ap^R^ was low for all mutants tested with the exception of the mutants obtained for FlgB F45Y and F45W. We were initially surprised to find that following marker rescue, many of the mutants were not able to grow on Ara-5μg/ml Ap plates, which they were originally selected on. However, this should not have been unexpected given the low concentration of Ap used in the selective media and the high number of colonies (~5,000) plated from the Tc^S^ selection plates onto Ara-5μg/ml Ap screening plates.

Most *flhB* mutants presented in Table 6 allowed for the secretion of the FlgB-Bla allele expressed from P_*araBAD*_ (Ara-Ap^R^), but with reduced secretion of the wild type FlgB, as measured by the Lac phenotypes without arabinose. Ara-Ap^R^ mutants obtained with FlgB-Bla F45Y and F45W substitutions exhibited similar phenotypes to the wild type parent strain (TH27282), while mutants obtained with FlgB-Bla F45A, F45S, F45T and F45R allowed FlgB-Bla to be secreted, but resulted in the inability to construct a functional HBB (decreased *fljB*::MudJ expression without arabinose). The parent strains (containing *flhB* wild type) for FlgB-Bla F45A (TH27277), F45S (TH27289), F45T (TH27277) and F45R (TH27285) did not allow any secretion of the FlgB-Bla allele and were completely Ara-Lac^+^ (Table 6 and Fig 4C).

Mutants obtained with FlgB-Bla F45L exhibited impaired HBB assembly with added arabinose (Ara-Lac^-^), but remained Ara-Ap^S^. One possibility is that FlgB-Bla F45L is stuck in the secretion apparatus blocking both HBB assembly and FlgB-Bla secretion (Fig 4E). This could be due to a stronger interaction of the FlgB-Bla F45L to the mutant FlhB motif and supports the hypothesis of Bryant et al that early substrates are recognized through interaction with the FlhB_CCD_ hydrophobic pocket [49].

We found one allele in *flhB* (A286P A341A L344H) that impaired FlgB secretion (Lac^-^ without arabinose; Fig 4F), but yet permitted FlgB-Bla F45Y to be secreted to higher levels of Ap^R^ than in the *flhB* parent strain. This mutant (TH27523) demonstrates that it is possible to alter the FlhB hydrophobic pocket and allow a different secretion-substrate to be selectively exported. We were surprised that this mutant prevented the secretion of FlgB F45 and allowed F45Y to be secreted, given the similarity between phenylalanine and tyrosine residues. In order to ensure that *flhB* (A286P A341A L344H) was impaired for wild type FlgB secretion, this *flhB* mutation was moved into the FlgB-Bla wild type parent strain (TH27382), and tested for Ap. The wild type FlgB-Bla was not secreted upon addition of arabinose (Ara-Ap^S^), demonstrating that the FlhB (A286P A341A L344H) mutant selectively impaired FlgB wild type secretion (Table 7, see TH27282 and TH27545 phenotypes).

**Table 7:** Phenotypes of selected mutants in *flhB* in combination with specific *flgB-bla* fusions expressed from P_*araBAD*_ with amino acid substitutions at codon 45 of *flgB*.

Strain^a^	FlgB^b^	Lac^c^		PPBS-Ara-Ap^d^						FlhB sequence^e^		
		no ara	* *ara	5	7.5	10	15	30	50	A286 (gcg)	A341 (gcg)	L344 (ttg)
TH27283	**F45Y**	++++	++++	+	+/-	-	-	-	-	*wild type flhB*		
TH27523	**F45Y**	-	-	+	+	+	-	-	-	P (ccg)	A (gct)	H (cat)
TH27282	**F45**	++++	-	+	+	+	+	+	-	*wild type flhB*		
TH27545	**F45**	-	-	-	-	-	-	-	-	P (ccg)	A (gct)	H (cat)
TH27285	**F45R**	++++	++++	-	-	-	-	-	-	*wild type flhB*		
TH27538	**F45R**	++	-	+	+/-	-	-	-	-	A (gca)	V(gtc)	E (gag)
TH27549	**F45R**	++	-	+	-	-	-	-	-	A (gcg)	A (gcg)	E (gag)
TH27548 (flhD*C*)	**F45R**	++++	++++	+	+	+	+	-	-	A (gcg)	A (gcg)	E (gag)
TH27287	**F45E**	++++	++++	-	-	-	-	-	-	*wild type flhB*		
TH27547 (flhD*C*)	**F45E**	-	-	+	+/-	-	-	-	-	A (gcg)	A (gcg)	R(cgt)
TH27562	**F45E**	-	-	-	-	-	-	-	-	A (gcg)	A (gcg)	R(cgt)

^a^All strains had the following genotype: Δ*araBAD*::(*flgB*-5’UTR)-*flgB-bla* (P_*araBAD*_-*flgB*-*bla*) *fljB5001*::MudJ Δ*hin-5718*::FRT and FlhB A286NNN A341VNN L344VNN, where amino acid substitutions at codon 45 of the P_*araBAD*_-*flgB*-*bla* constructs are indicated and the *flhB* mutation reported. The detailed genotype is listed on S6 Table.

^b^Indication of the amino acid substitution at position 45 of FlgB expressed from P_*araBAD*_-*flgB*-*bla*

^c^Flagellar class 3 σ^28^-dependent transcription of a *fljB*::MudJ reporter fusion construct (*fljB5001*::MudJ Δ*hin-5718*::FRT) was determined on Mac-Lac and TTC-lac indicator medium with and without added arabinose (++++: TTC-Lac white and ML dark red; +++: TTC-Lac pink and ML dark red; ++: TTC-Lac red and Mac-Lac red; +: TTC-Lac dark red and ML pink; —: Mac-Lac white)

^d^Ampicillin resistance was tested on PPBS- plates with and without arabinose (PPBS-Ap5) and with varying ampicillin concentrations as shown in μg/ml Ap. None of the strains grew on PPBS-Ap5 without arabinose. (+: grew well; +/-: grew a little; -: did not grow)

^e^*flhB* mutations, as obtained by DNA sequence analysis

An interesting mutant in *flhB* was FlhB (A286A A341V L344E) that permitted FlgB-Bla F45R to be secreted more than in the wild-type FlhB (A286 A341 L344). This mutant affected wild type FlgB secretion, as seen with the decrease of Lac activity without arabinose. The single L344E substitution in FlhB was constructed by λ-Red recombineering and was found to still permit FlgB-Bla F45R secretion (TH27549; Table 7). The introduction of protease-resistant alleles of the flagellar master regulatory genes *flhD flhC* (flhD*C*) that are resistant to ClpXP proteolysis and increase overall flagellar gene expression resulted in increased FlgB-Bla F45R secretion levels, as expected (see TH27448; Table 7). The FlhD and FlhC proteins combine to form the FlhD_4_C_2_ transcriptional activator complex of flagella HBB class2 gene transcription [54]. FlhD and FlhC are regulated at a post-transcriptional level by the ClpXP protease through YdiV/RflP(for FlhD) and FliT(for FlhC) adaptor proteins that target FlhD and FlhC to ClpXP [55, 56]. We reasoned that since FlhB L344E allowed some FlgB-Bla F45R secretion, L344R might permit secretion of FlgB-Bla F45E. The construct was made, but the FlhB L344R allele was defective for the secretion of both wild type F45 and mutant F45R alleles, although a low level of secretion was observed in the flhD*C* background (Table 7, strains TH27562 and TH27547).

Of note, identical substitutions in FlhB (A286V A341A L344V) were isolated as able to secrete F45W FlgB-Bla and F45Y FlgB-Bla (see TH27522 and TH27526- Table 6). This was not surprising given the similarity between the tryptophan and tyrosine residues. FlhB (A286A A341R L344L) was also independently isolated as able to secrete FlgB-Bla with either F45A or F45C substitutions (see TH27531 and TH27537- Table 6).

All together, these data strongly support the hypothesis that an interaction of position 45 of FlgB with amino acids in the conserved hydrophobic pocket of the FlhB_CCD_ occurs during secretion as amino acid substitutions in the hydrophobic pocket allow for the secretion of FlgB-Bla substrates carrying position 45 substitutions that are not secreted by a flagellar T3S system with a wild-type FlhB protein.

## Discussion

The goal of this research was to characterize possible amino acid secretion signals in the early flagellar T3S substrates FlgB and FlgC. It had been proposed that F45 of FlgB and F49 of FlgC were important for recognition by a hydrophobic pocket in the FlhB component of the flagellar T3S system [48]. Rather that targeting those residues specifically, we decided to identify regions of FlgB and FlgC important for their secretion and determine if a forward genetic approach would independently reveal F45 of FlgB and F49 of FlgC to be important for their secretion. Our system used a *lac* operon fusion to a σ^28^-dependent flagellar class 3 promoter (*fljB*::MudJ) as an indicator for the presence of a functional flagellar T3S apparatus. A defective apparatus would be unable to secrete the anti-σ^28^ factor FlgM and result in a Lac^-^ phenotype on lactose indicator medium. Our system also employed fusion of β-lactamase, deleted for its Sec-dependent secretion signal (Bla), as a reporter for secretion of flagellar secreted substrates into the periplasm. Resistance to ampicillin (Ap^R^) requires that the Bla fusions be secreted by a functional flagellar T3S system into the periplasmic space where the Bla reporter will fold into an active conformation in order to confer Ap^R^.

Targeted deletions of *flgB* and *flgC* revealed three segments of FlgB, amino acids 9 through 18, 39 through 48, and 49 through 58, which when deleted exhibited a significant reduction in FlgB-Bla secretion levels as indicated by reduced resistance to Ap. For FlgC, only a deletion of amino 6 though 14 showed a significant reduction in Ap resistance levels. A doped oligonucleotide mutagenesis for the amino acid 39 through 48 region of FlgB revealed position F45 to be critical for secretion as had been predicted earlier. However, a targeted mutagenesis of F49 of FlgC revealed this position, although critical for assembly, was not as critical for FlgC secretion.

We characterized residues in the FlhB_CCD_ that form a hydrophobic patch predicted to interact with F45 of FlgB for secretion. We determined that these residues were important for FlgB-Bla secretion. L344 is a critical residue although isoleucine and valine at this position also produced wild-type secretion levels. Valine or serine substitutions at A286 also resulted in wild-type secretion while A341 was less restrictive as substitutions to glycine, valine or serine resulted in wild-type FlgB-Bla secretion levels in our assay system. A codon randomization for positions 286, 341 and 344 of the FlhB_CCD_ hydrophobic patch was used to identify amino acid substitutions that would allow for secretion of secretion-defective FlgB-Bla fusions carrying amino acid substitution alleles at position 45. Suppression of secretion-defective alleles was possible by amino acid changes in the FlhB_CCD_ hydrophobic patch but not to wild-type levels of secretion.

In conclusion, we did not find an early flagellar T3S signal that was conserved in amino acid sequence within the N-terminus FlgB and FlgC. However, this work agrees with an earlier study that identified a hydrophobic patch in the FlhB_CCD_ that was shown to be important for secretion of the early flagellar FlgE (hook) and FlgD (hook-cap) secretion substrates [48]. The prediction that F45 of FlgB is an important residue in targeting FlgB for secretion was found to be the case, but not for F49 of FlgC. A recent study by Bryant et al. characterized the early secretion signal for early secretion substrate FlgD [49]. They identified residue F36 of FlgD as important for secretion. These authors also identified residues 2 through 5 of FlgD as critical for FlgD secretion. These results are similar to what we observed for FlgB suggesting a common early secretion signal between the FlgB and FlgD substrates. The work presented in this study identified two regions, amino acids 9 through 18 of FlgB and 6 through 14 of FlgC, that appear to be important for secretion, which may function similarly in FlgB and FlgC secretion to residues 2–5 in FlgD. We agree with the proposal of Bryant et al. that early flagellar T3S-substrates have two targeting signals in the N-terminal peptide region that appear to be separated by a spacer sequence. However, 10 amino acids deletions between the two regions in the N-terminus of FlgB or and FlgC that appear to encode signals important for secretion did not affect secretion so the jury is still out. We suspect that the strength of one signal determines the strength of another. By this we mean that, for FlgC, the interaction of the amino acid 5–14 is of sufficient strength that the interaction with the region including F49 is less important. The reverse may then be true for FlgB, that the F45 interaction is strong resulting a less stringent requirement for interaction with residues in the region including amino acids 9–18 (Fig 2). Future studies that target these specific regions are still needed to fully characterize important amino acid residues in the N-terminus of FlgB and FlgC for their secretion as early flagellar T3S substrates.

## Materials and methods

### Bacterial strains, media, and standard genetic techniques

Strains used in this study are listed in S6 Table. All strains were derived from *Salmonella enterica* serovar Typhimurium strain LT2. Lysis broth (LB; 10g tryptone, 5g yeast extract, 5g NaCl per liter)) was used as a rich medium for growing bacterial cultures. Antibiotics were added as necessary using final concentrations of 5 to 200 μg/ml sodium ampicillin, 12.5 μg/ml of chloramphenicol, 50 μg/ml of kanamycin sulfate and 15 μg/ml tetracycline-HCl. Arabinose (Ara) was used at a 0.2% final concentration to induce transcription from the *araBAD* promoter (P_*araBAD*_). Motility assays were performed in soft agar motility plates (per liter: 10g tryptone, 5g NaCl, 3g Bacto agar). Minimal E salts medium supplemented with 0.2% glucose was used as a minimal medium [57]. E minimal medium with carbon sources other than glucose was no-carbon E (NCE) medium that lacks citrate. Phenotypic lactose activity was observed using MacConkey lactose indicator medium (Mac-Lac), or 3,5-triphenyltetrazolium chloride-lactose (TTC-Lac) indicator medium supplemented with 0.2% arabinose if required [58]. Selection of tetracycline-sensitive (Tc^S^) clones was done on zinc-fusaric acid selection plates [59]. Proteose peptone bile salt (PPBS) plates contained per liter: 17g Bacto peptone, 3g Bacto proteose peptone, 5g NaCl, 12 g Apex agar and 1.5g Difco bile salt #3. PPBS plates were used to quantify resistance to varying levels of ampicillin on solid medium. The generalized transducing phage P22 was used for all transductional crosses [60]. λ-Red-based recombineering was used for all targeted chromosomal mutagenesis [59]. Proof reading polymerase (Phusion) was used for the preparation of all the DNA fragments used in chromosomal DNA targeting experiments via λ-Red. All oligonucleotides, listed in S7 Table, were synthesized at Eton Biosciences, except for the doped oligo nucleotides that were synthetized at the core facility of the University of Utah. All constructs were confirmed by DNA sequencing analysis (Eton Biosciences).

### β-Lactamase fusions construction

The construction of C-terminal β-lactamase fusions to FlgB, FlgC, FlgF, FlgG and FlgJ was done by insertion of a *tetRA* element before the corresponding gene stop codon, via λ-Red recombination. The *tetRA* element was then replaced via λ-Red recombination followed by incubation on Tc^S^ selection plates with a DNA sequence that included the *bla* coding sequence lacking its first 23 amino acid codons, which remove the Sec secretion signal for Bla.

### Selection for expression of *flgB-bla* at the *araBAD* locus

The *araBAD* coding region was deleted and replaced with a *flgB-bla* fusion in order to place FlgB-Bla expression under arabinose induction by P_*araBAD*_ [46]. However, no FlgB-Bla was detected when the strain was grown in the presence of arabinose inducer. We suspected that the inability to produce FlgB-Bla expressed at the *araBAD* chromosomal locus might be due to an inability to initiate translation of the *flgB-bla* coding sequence due to the presence of the *araBAD* 5’UTR sequence. The predicted mRNA 2° structure for the *araBAD* 5’UTR preceding the first 10 codons of *flgB* shows a strong stem loop structure that could prevent the predicted ribosome binding sequence from translation initiation (S1 Fig). A deletion of the *mutS* was introduced into the P_*araBAD*_-*flgB*-*bla* strain gene to increase the frequency of rare base substitution alleles in the 5’UTR of *flgB* and the resulting construct was plated on arabinose plates containing 15 μg/ml ampicillin (Ara-Ap plates). Ampicillin-resistant (Ap^R^) colonies arose at a high frequency (10^−5^). Ten independent colonies were picked and DNA sequence analysis revealed three different base substitutions in the 5’-UTR and codons 6 and 9 of *flgB* that are predicted to disrupt the mRNA 2° structure and presumably allow access of the ribosome for translation of *flgB-bla* (S1 Fig). The P_*araBAD*_-*flgB*-*bla* was reconstructed with both the 5’UTR of the *araBAD* operon and the coding region replaced with the 5’UTR of *flgB* and the *flgB* coding sequence (P_*araBAD*_-(5’UTR-*flgB*)-*flgB*-*bla*). This construct produced an MIC level for Ap of 25 μg/ml (S1 Table) and grew on Ara-Ap (15 μg/ml) plates. All further experiments utilized the P_*araBAD*_-(5’UTR-*flgB*)-*flgB*-*bla* construct. The predicted mRNA 2° structure of the *flgB* 5’-UTR preceding the first 10 codons of *flgB* exhibits a folded pattern that would not occlude the ribosome binding site or the AUG start codon (S1 Fig). Expression of either *flgB*-*bla* or *flgC*-*bla* expressed at the chromosomal *flg* loci resulted in MIC values for Ap of 100 μg/ml (S1 Table). The higher MIC levels when expressed from the chromosomal *flg* loci suggested that other factors such as transcription or mRNA stability contribute to increased *flgB*-*bla* expression from its native locus.

### Deletions of 10 amino-acid codon segments in FlgB-Bla and FlgC-Bla constructs

The 10 amino-acids deletion constructs were produced in strains expressing either FlgB-Bla (TH23899) or FlgC-Bla (TH23902). The strategy to produce in-frame deletions in FlgB and FlgC is summarized in S2 Fig. *TetRA* elements were inserted at positions separated by 20 codons of the gene starting from the 3’-end of the coding sequence (S2A Fig). Each *tetRA* element was then used to construct the 10 amino acid deletion before (S2B Fig) or after (S2C Fig) the site of *tetRA* element insertion using a DNA fragment deleted for the region of interest via λ-Red recombination followed by Tc^s^ selection. The DNA fragment deleting the 10 amino acid codons was produced using a 4-cycle fill-in reaction between a forward oligonucleotide, containing 40bp homology to the sequence upstream of the 10 amino acid deletion, and 15bp sequence of the sequence after the deletion, and a reverse primer containing 40-50bp of the sequence after the deletion. All deletions were confirmed by DNA sequence analysis.

### Targeted mutagenesis of FlgB

Two *tetRA* elements were introduced in FlgB, deleting amino acids 39 through 48 and amino acids 49 through 58 of FlgB, respectively, in strain TH7365 (*fljB5001*::MudJ Δ*hin-5718*::FRT) containing the *lacZ* reporter to the class 3 promoter of the *fljBA* operon. The *tetRA* elements were then replaced, via λ-Red recombination and Tc^S^ selection, with PCR fragments containing at least 40 bp of homology on each side of the site of *tetRA* insertion and a doped region introducing base substitutions in *flgB* amino acid codons 39 through 48 and 49 through 58, respectively. The “doped” oligonucleotides were synthesized so that the chemical mixture for each wild-type base contained a small amount of the three other bases such that on average each oligonucleotide contained one single random base substitution mutation throughout the coding sequence being targeted for mutagenesis. The doped oligo nucleotides were designed so that it contained a 18bp of non-doped sequence at its 5’ and 3’ends (lower case primers #8100 and #8101- S7 Table). The doped oligo nucleotides were then used as a template in a 10-cycle PCR reaction using forward and reverse external primers adding the homology for the recombination (primers #8096 and #8097 for doped oligo #8100 and #8098 and #8099 for doped oligo #8101– S7 Table). Tc^S^ recombinants were then screened on Mac-Lac indicator plates for flagellar class 3 promoter expression. DNA sequence analysis revealed base changes in *flgB* that resulted in amino acid substitutions.

### Random mutagenesis of amino acid codons F45 of FlgB and F49 of FlgC

A *tetRA* element was introduced in *flgB*, so that it replaced amino acid codon F45 of FlgB via λ-Red recombination. An oligo nucleotide (#8316) containing NNN at the codon 45 position of *flgB* sequence was synthesized and used to form a primer-dimer with a reverse primer containing homology after codon 45 of *flgB* (#8097). A 4-cycle fill-in reaction was set to fill the ends of the DNA fragment containing homology before and after codon 45 of *flgB* and NNN instead of the F45 codon. The resulting DNA fragment was introduced via λ-Red recombineering, using Tc^S^ selection. Alleles coding for all twenty amino acids at codon 45 of *flgB* were obtained, tested for hook basal body assembly using expression of the *fljB*::MudJ as a reporter as described above. The same strategy was used to introduce codons for all twenty amino acids at the *flgC* F49 codon position using primers #9219 and #9218 to produce a double stranded DNA with NNN in place of codon 49 of FlgC to replace the *tetRA* element in strain TH26845 (pSim5/ *flgC8166*::*tetRA*(ΔAA49::*tetRA*) *fljB*5001::MudJ Δ*hin-5718*::FRT).

### Construction of *flgB-bla* or *flgC-bla* reporters expressed from P_*araBAD*_ that contain codon substitution alleles

The genomic DNA from *flgB* alleles was purified and used as a template for creating PCR fragments with homology to the beginning of *flgB* and *bla*, using primers #7851 and #1750. These PCR fragments were then used to replace a *tetRA* element in *flgB-bla* expressed from P_*araBAD*_ at the chromosomal *araBAD* locus in strain TH25008(pKD46 /Δ*araBAD*2097::5’UTR*flgB* -*flgB*::*tetRA*(after AA48)-*flgB-bla* Δ*flgBC*6557), using λ-Red recombination followed by Tc^S^ selection. The *flgC* alleles were moved to the arabinose locus using TH23889 (pSim5/Δ*araBAD1066*::*tetRA*-*bla* Δ*flgBC6557*) as the recipient strain and primers #3822 and #1750.

### Simultaneous mutagenesis at codons A286, A341 and L344 of *flhB*

A *tetRA* element designed to delete codons A286 through L344 of *flhB* was first inserted in *flhB* in strain TH7365 (*fljB5001*::MudJ Δ*hin-5718*::FRT). Oligonucleotides containing VNN at codons A286, A341 and L344 of FlhB were synthetized, where N corresponds to the incorporation of either adenine (dATP), cytosine (dCTP), guanine (dGTP), or thymine (dTTP), and where V corresponds to the incorporation of either adenine (dATP), cytosine (dCTP) or guanine (dGTP). A DNA fragment containing A286VNN A341VNN L344VNN substitution mutations in *flhB* was produced as illustrated in S3 Fig. Double stranded DNA fragments were produced that contained *flhB* A286VNN for fillin-1 (using primers 9460 and 9368) and *flhB* A341VNN and L344VNN for fillin-2 (using primers 9369 and 8821), using a 4-cycle fill-in reaction. A DNA fragment was also produced using genomic DNA from strain LT2 and primers 9428 and 9427 so that it had some homology sequence with fill-in 1 and fillin-2 (see S3 Fig). The 3 DNA fragments were mixed together without primers followed by a 10-cycle PCR reaction, allowing the 3 fragments to stitch the fragments together. A final 15-cycle PCR reaction on the stitched product, using external primers 9474 and 9571 was used for the λ-Red substrate product used to replace *flhB*::*tetRA* (ΔAA286-344). Colonies from 30 Tc^S^ selection plates (each containing approximately 5,000 colonies) were pooled together giving a pool of cells of approximately 150,000 Tc^S^ colonies. The pool (CD60) was frozen at -80°C until further use.

To ensure that our method resulted in targeted mutagenesis of codons 286, 341 and 344 of *flhB*, a dozen Tc^S^ mutants were chosen at random and the DNA sequence for this region of *flhB* was obtained. The DNA sequence (S3 Table) showed that the method worked well, resulting in a variety of codon substitutions at the specified positions in *flhB*. The selection strain carried a *fljB*::MudJ reporter to allow for selection of a functional FlhB protein, which is required to allow for FlgM secretion and a Lac^+^ growth phenotype. The pooled cells of Tc^S^ mutants (CD60) were plated onto minimal-lactose selective media in order to determine the prevalence of functional FlhB. Surprisingly, a high number of cells (1 out of 25) from the Tc^S^ pool grew on the lactose selection plates. This result suggested that the residues in the surface-exposed hydrophobic pocket in the FlhB_CCD_ were not under a strict selection for FlhB function. Twenty-one Lac^+^ colonies were sequenced for *flhB*. Residues 286 and 341 could tolerate multiple, different amino acid residues to produce functional FlhB, whereas residue 344, appeared more critical for FlhB function. Only codons for the wild-type L344 residue or the L344I substitution were obtained that resulted in a wild-type FlhB phenotype (S4 Table).

The NCE-Lac^+^ colonies were pooled together and frozen at -80°C until needed (CD74). We noticed a large variation of Lac^+^ phenotypes on Mac-Lac and TTC-Lac indicator plates from this pool. In order to enrich for Lac^+^ colonies with the highest levels of *lac* operon expression, the NCE-Lac^+^ CD74 cell pool was grown overnight and 0.5ml of a 10^−7^ dilution of this overnight culture was plated onto NCE-Lac plates at 37°C. The NCE-Lac plate was replica printed onto TTC-Lac plates and a thousand of the white colonies (the Lac^+++^ mutants) were patched uniformly on LB plates and incubated overnight at 37°C. These colonies were pooled together, vortexed well, diluted to ~10^9^ cell/ml and stored as pool CD75.

### Amplicon sequencing analysis

A portion of Tc^S^ pool CD60 was collected and grown overnight at 37°C with aeration. A 0.4 ml portion of the 10^−6^ dilution of the overnight culture (approximately 2,000 cells) was added to 1.6 ml of LB media and grown overnight at 37°C. To ensure that the number of cells was around 2,000 cells, the 10^−6^ dilution culture was further diluted 10-fold and 0.4 ml of the 10^−7^ dilution was plated on LB plates and incubated overnight. The next morning, the number of colonies counted on the LB plate was 257. The genomic DNA was extracted from the 10^−6^ dilution culture and used as a template for amplicon sequencing analysis. A portion of the Lac^+++^ pool CD75, which contained a thousand of the highest Lac^+^ expression colonies, was grown overnight at 37°C with aeration. The genomic DNA was extracted and used as a template for amplicon sequencing analysis. Primers containing Illumina adaptor sequences and priming sequence to the C-terminus of *flhB* (#9631 and #9632) were used to amplify the *flhB* region of interest from the genomic DNA of the pooled mutants. A 15-cycle PCR was then performed on the pool with KAPA HiFi DNA polymerase. The PCR fragments were purified and sent to next generation sequencing analysis (Genewiz/Azenta). Overlapping reads were combined for each read pair. For disagreement between the base calls in the overlapping region, the base with the higher quality score was used if the quality score was at least 10. Otherwise, the read pair was discarded. Read pairs with insertions, deletions, or mutations in the nonrandomized regions were also discarded. For the 1,000 sequences supported by the most read pairs in each pool, the number of each amino acid at each position was counted. The log odds score was calculated (log2(Lac^+^ count/total Tc^S^ count)) for each amino acid at each position, and the statistical significance was determined using Fisher’s exact test.

### β-lactamase (Bla) secretion assays

For the liquid assays to determine the minimal inhibitory concentration (MIC) for levels of ampicillin resistance (Ap^R^), strains were inoculated from single colonies at 37°C and incubated overnight with aeration in LB media in the presence of arabinose if required. The overnight cultures were diluted 200-fold in buffered saline and the cells were diluted 100-fold in 96-well plates (2ul in 200ul) in LB medium containing appropriate concentrations of ampicillin in the presence of arabinose if required. The 96-well plates were grown overnight at 30°C with aeration, and minimal inhibitory concentration (MIC) was determined after an 18-hr incubation period as the lowest Ap concentration at which the cells did not grow. For the plate Ap^R^ assays, strains were inoculated from single colonies at 37°C overnight with aeration, in LB media, in the presence of arabinose if needed. The overnight cultures were diluted 100-fold into 2 ml of LB supplemented with arabinose if necessary, and grown for 90 min at 37°C with aeration. Cells were then diluted a 1,000-fold into buffered saline. Aliquots of 4 μl of the diluted cell cultures were spotted onto PPBS plates with varying concentrations of Ap that were supplemented with arabinose if needed. The PPBS-Ap plates were incubated overnight at 37°C. Growth on PPBS-Ap plates with varying Ap levels are reported. For each assay, a minimum of 3 independent cultures were tested. For the liquid assays, a stock solution of 100 mg/ml of ampicillin was prepared in water, aliquoted and kept at -20°C. One single aliquot was used for each assay avoiding freeze thawing. For the PPBS-Ap plates, stock solutions of 6 or 20 mg/ml of ampicillin were prepared in 50% ethanol and kept at -20°C. Stock solutions of sodium ampicillin were made fresh every 3 months.

### Western blot quantification of FlgB-Bla and FlgC-Bla expression levels

Cells were grown overnight in LB with arabinose at 37°C. Cell pellets were resuspended in SDS-buffer containing β-mercaptoethanol and boiled for 4 min. Proteins were separated on a 12% SDS-PAGE and transferred on to polyvinylidene fluoride (PVDF) membranes. Immunoblotting to detect FlgB-Bla fusion levels was performed using a mouse monoclonal anti-β-lactamase antibody (8A5.A10 Abcam) and secondary Licor antibody (IRDye 800CW Goat anti-mouse 926–32210). Infrared signals were detected using an Odyssey Infrared Imaging System.

### Screening for suppressors in the hydrophobic pocket of FlhB allowing *flgB-bla* alleles secretion

To isolate specific suppressor alleles located to the hydrophobic pocket of FlhB, P22 grown on the pool of targeted FlhB_CCD_ substitutions (CD60) was used to move the *flhB* region into strains expressing *flgB-bla* F45 codon substitution alleles expressed from the *araBAD* locus (P_*araBAD*_-*flgB-bla*) that contained a *tetRA* element deleted for codons 286–344 of *flhB*. The Tc^S^ transductants were pooled together. A small portion of the pool was grown in LB with arabinose overnight and then plated onto PPBS-Ap5-Ara plates. Ap^R^ colonies were purified and tested on Mac-Lac, Mac-Lac-Ara, and PPBS-Ap and PPBS-Ap-Ara plates. Mutants that were Ap^R^ without added arabinose were discarded. Select mutants were marker rescued by using either transduction or λ-Red to move the *flhB* mutant allele back into the original strain background expressing FlgB F45 substitution alleles expressed from P_*araBAD*_ that also contained the *tetRA* element deleting codons 286–344 of *flhB*.

## Supporting information

S1 Fig Predicted mRNA 2° structure for the *araBAD* 5’UTR preceding the first 10 codons of *flgB* shows a strong stem loop structure that could prevent the predicted ribosome binding sequence from translation initiation. (TIF)

S2 Fig Strategy to produce clean deletions in *flgB* and *flgC*. *TetRA* elements were inserted every 20 amino acids of the genes starting from the end (A). Each *tetRA* element was then used to produce the 10 amino acid codon deletion before (B) or after (C) the *tetRA* element.
(TIF)

S3 Fig Strategy to produce the *flhB* A286VNN A341VNN L344VNN λred fragment. Fill-in fragments were produced that contained *flhB* A286VNN for fillin-1 and *flhB* A341VNN L344VNN for fillin-2 (Panel A). The DNA between the fill-in fragments was amplified using genomic DNA of LT2 and primers so that the 3 DNA fragments could recombine during the stitching step (Panel B). The whole fragment was then amplified using end primers (Panel C).
(TIF)

S4 Fig Expression levels of selected *flgB* F45 and *flgC* F49 alleles expressed from the *araBAD* locus (P_*araBAD*_-*flgB-bla* or P_*araBAD*_-*flgC-bla*), using anti-beta-lactamase antibodies. (TIF)

S1 Table MIC values for early flagellar secretion substrates fused to β-lactamase. (DOCX)

S2 Table Single *flgB* mutants isolated through doped oligo mutagenesis of *flgB*. Class 3 gene expression activity phenotypes, motility and secretion assay of selected alleles.
(DOCX)

S3 Table Randomization of codon F45 in *flgB* and class 3 activity phenotypes, motility and secretion assays. (DOCX)

S4 Table Mutagenesis of the FlhB C-terminus surface exposed hydrophobic pocket (FlhB A286NNN A341VNN L344VNN) in a *fljB5001*::MudJ Δ*hin-5718*::FRT background to determine the effect of *flhB* mutations on HBB assembly. (DOCX)

S5 Table Searching for specific alleles in *flhB* that allow secretion of FlgB-Bla fusions expressed from P_*araBAD*_ with amino acid substitutions at codon 45 of *flgB*. The FlhB A286NNN A341VNN L344VNN pool was transduced into several *flgB* mutant strains expressing *flgB-bla* alleles from P_*araBAD*_, and screened for secretion on PPBS-Ara-Ap plates.
(DOCX)

S6 Table List of strains used in this study. (DOCX)

S7 Table List of primers used in this study. (DOCX)
