# Supplementary figures and images for: Targeting early proximal-rod component substrate FlgB to FlhB for flagellar-type III secretion in Salmonella
Source: PLoS Genet. 2022 Jul 12;18(7):e1010313. doi: 10.1371/journal.pgen.1010313 (PMC9307174; doi:10.1371/journal.pgen.1010313)

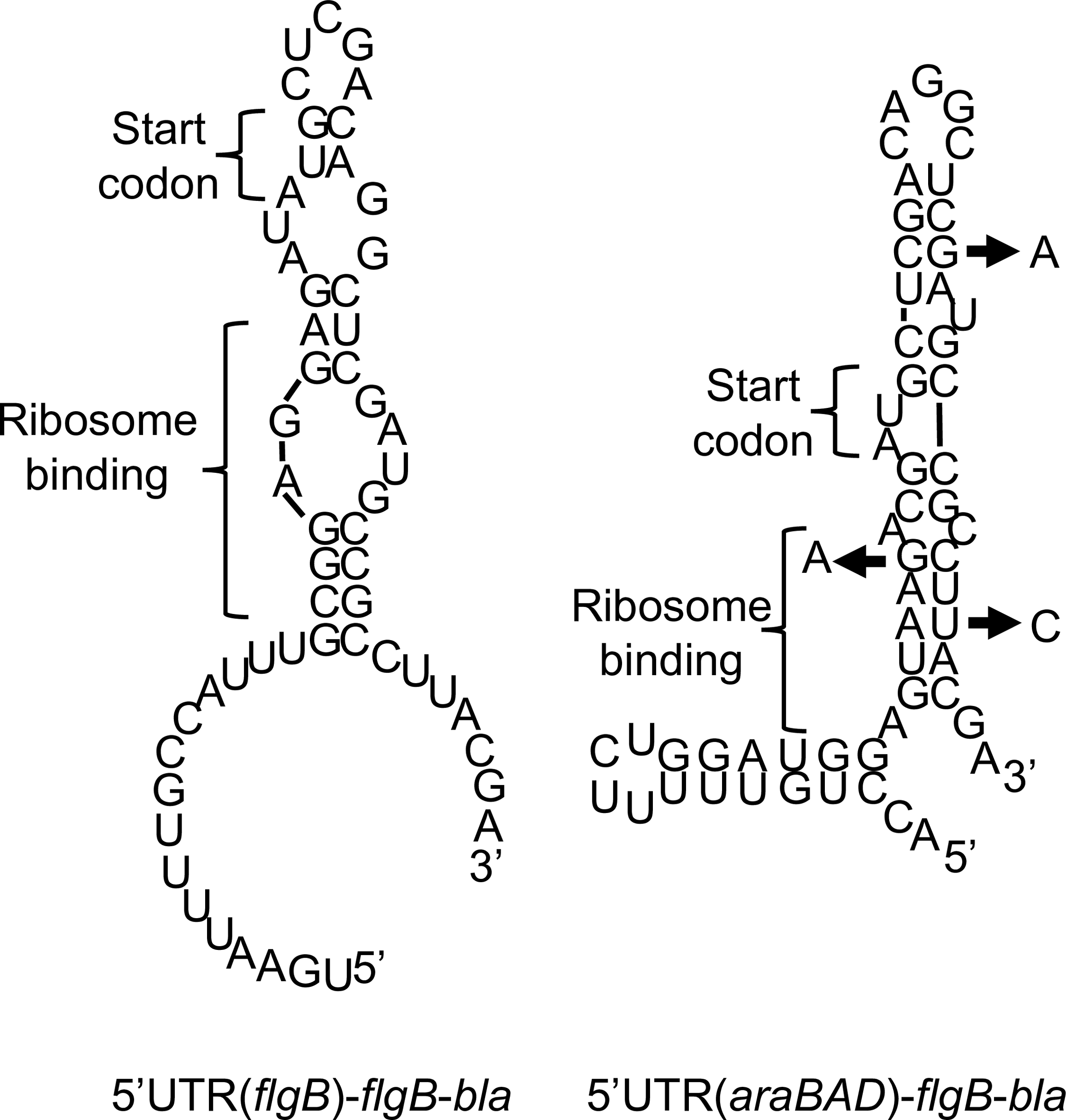

Supplement: S1 Fig — (TIF) [file pgen.1010313.s001.tif]

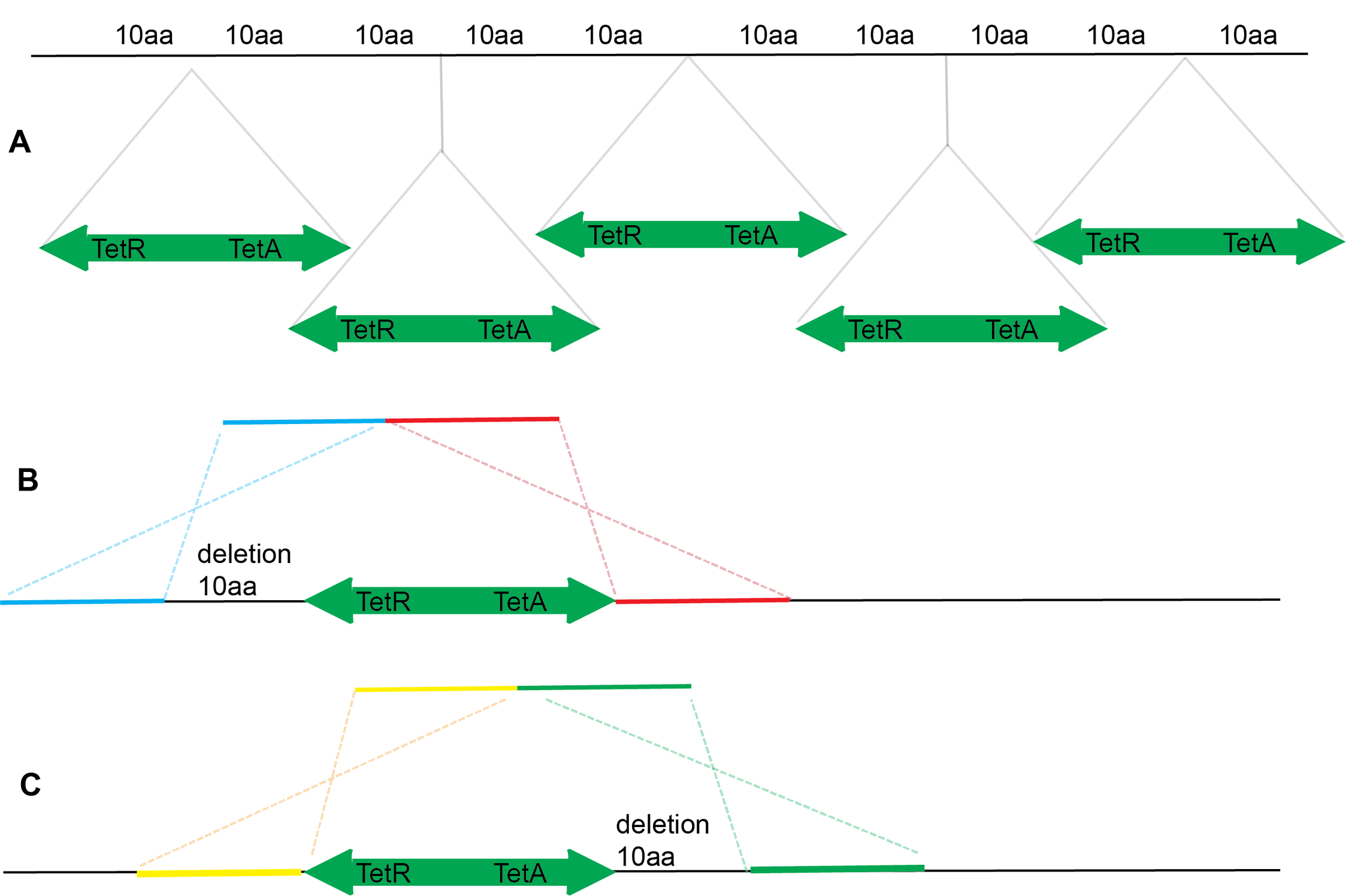

Supplement: S2 Fig — TetRA elements were inserted every 20 amino acids of the genes starting from the end (A). Each tetRA element was then used to produce the 10 amino acid codon deletion before (B) or after (C) the tetRA element. (TIF) [file pgen.1010313.s002.tif]

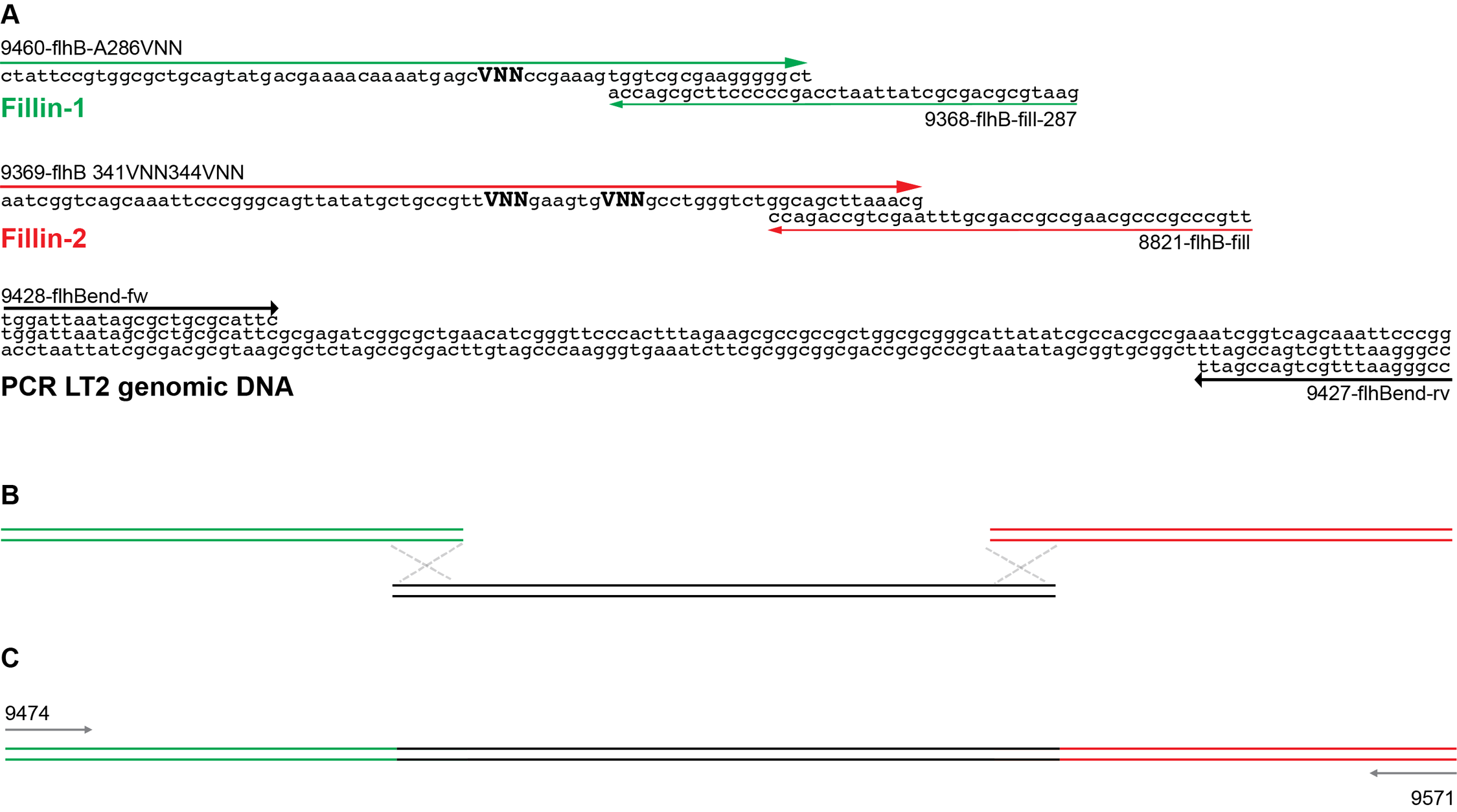

Supplement: S3 Fig — Fill-in fragments were produced that contained flhB A286VNN for fillin-1 and flhB A341VNN L344VNN for fillin-2 (Panel A). The DNA between the fill-in fragments was amplified using genomic DNA of LT2 and primers so that the 3 DNA fragments could recombine during the stitching step (Panel B). The whole fragment was then amplified using end primers (Panel C). (TIF) [file pgen.1010313.s003.tif]

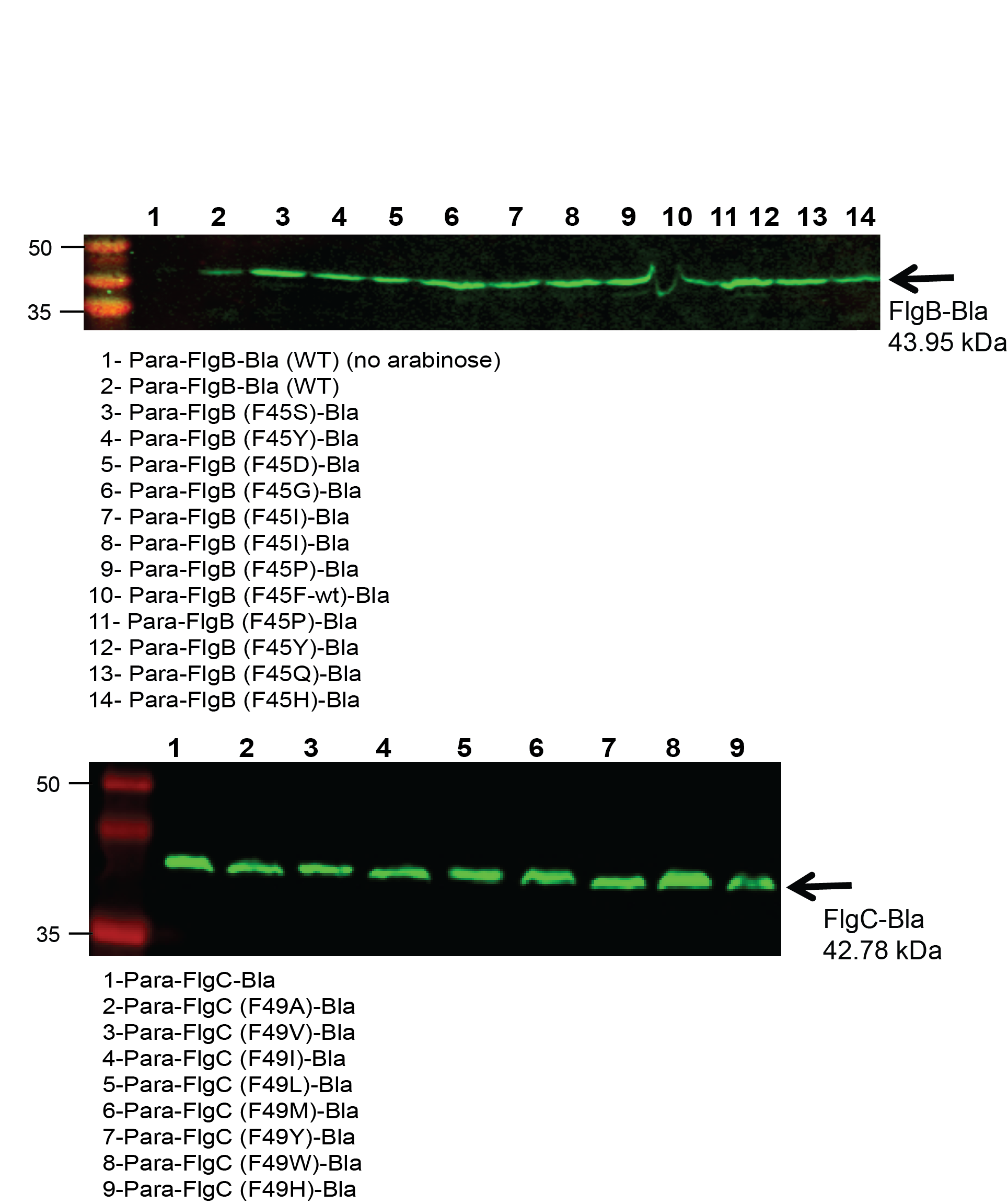

Supplement: S4 Fig — (TIF) [file pgen.1010313.s004.tif]
