# Supplementary material for: Targeting early proximal-rod component substrate FlgB to FlhB for flagellar-type III secretion in Salmonella
Source: PLoS Genet. 2022 Jul 12;18(7):e1010313. doi: 10.1371/journal.pgen.1010313 (PMC9307174; doi:10.1371/journal.pgen.1010313)
Supplement: S1 Table — (DOCX) [file pgen.1010313.s005.docx]

**S1 Table.** MIC values for early flagellar secretion substrates fused to β-lactamase.

|  |  | **MIC values (μg/ml)** | | |  | |
| --- | --- | --- | --- | --- | --- | --- |
|  |  | repeat 1 | repeat 2 | repeat 3 | |  |
| TH27139 | Δ*BAD2168::flgB-bla*(*araB* 5’UTR) Δ*flgBC* | 1.5 | 1.5 | 1.5 | |  |
| TH25004 | Δ*BAD2096::flgB-bla*(*flgB* 5’UTR) Δ*flgBC* | 25 | 25 | 25 | |  |
|  |  |  |  |  | |  |
| TH24148 | *flgJ8650::bla* | 25 | 25 | 25 | |  |
| TH24149 | *flgG8651::bla* | 50 | 50 | 50 | |  |
| TH24147 | *flgF8649::bla* | 100 | 100 | 50 | |  |
| TH23902 | *flgC8595::bla* | 100 | 100 | 100 | |  |
| TH23899 | *flgB8593::bla* | 100 | 100 | 100 | |  |

The *bla* coding lacking its Sec secretion signal was inserted before the stop codon of each indicated gene.

For strains TH27139 and TH25004, MIC’s were determined in the presence of arabinose.
