# Supplementary material for: Targeting early proximal-rod component substrate FlgB to FlhB for flagellar-type III secretion in Salmonella
Source: PLoS Genet. 2022 Jul 12;18(7):e1010313. doi: 10.1371/journal.pgen.1010313 (PMC9307174; doi:10.1371/journal.pgen.1010313)
Supplement: S2 Table — Class 3 gene expression activity phenotypes, motility and secretion assay of selected alleles. (DOCX) [file pgen.1010313.s006.docx]

**S2 Table.** Single *flgB* mutants isolated through doped oligo mutagenesis of *flgB*. Class 3 gene expression activity phenotypes, motility and secretion assay of selected alleles.

| **FlgB amino acid** | **Mutant number** | **Mutation^a^** | **Lac phenotype^b^** | **Motility^c^** | **ApR (PPBS plates)^d^** |
| --- | --- | --- | --- | --- | --- |
| 39 | AA39-48-60 | Q39L | ++++ | 100% | 75 |
| 40 | AA39-48-135 | A40E | ++ | 50% | 50 |
| 40 | AA39-48-57 | A40S | +++ | 75% | ND |
| 40 | AA39-48 1-11 | A40V | +++ | 75% | ND |
| 41 | AA39-48-34 | R41D | +/- | 10% | 75 |
| 42 | AA39-48-68 | D42Y | +/- | 10% | 75 |
| 42 | AA39-48-142 | D42Y | +/- | 10% | ND |
| 43 | AA39-48-65 | I43F | ++++ | 100% | 75 |
| 43 | AA39-48-19 | I43S | +/- | 5% | 100 |
| 43 | AA39-48-28 | I43S | +/- | 5% | ND |
| 43 | AA39-48-64 | I43S | +/- | 5% | ND |
| 44 | AA39-48-26 | D44G | +++ | 84% | 75 |
| 44 | AA39-48-25 | D44Y | +++ | 84% | 50 |
| 45 | AA39-48-21 | F45C | +/- | 8% | 5 |
| 45 | AA39-48-15 | F45I | +/- | 20% | 5 |
| 45 | AA39-48-120 | F45I | +/- | 20% | 5 |
| 45 | AA39-48-11 | F45L | +/- | 17% | 5 |
| 45 | AA39-48-16 | F45L | +/- | 17% | 5 |
| 45 | AA39-48-51 | F45L | +/- | 17% | 5 |
| 45 | AA39-48-96 | F45L | +/- | 17% | 5 |
| 45 | AA39-48-129 | F45L | +/- | 17% | 5 |
| 45 | AA39-48 1-4 | F45L | +/- | 17% | 5 |
| 45 | AA39-48 1-5 | F45M | +/- | 20% | 5 |
| 45 | AA39-48-37 | F45V | +/- | 34% | 5 |
| 45 | AA39-48-82 | F45V | +/- | 33% | 5 |
| 46 | AA39-48-127 | E46D | ++++ | 100% | ND |
| 47 | AA39-48-101 | S47C | ++++ | 100% | ND |
| 47 | AA39-48-105 | S47C | ++++ | 100% | ND |
| 47 | AA39-48-56 | S47I | +++ | 95% | 75 |
| 47 | AA39-48-140 | S47I | ++++ | 100% | ND |
| 47 | AA39-48-47 | S47R | ++++ | 100% | 100 |
| 48 | AA39-48-62 | E48D | +++ | 80% | 75 |
|  |  |  |  |  |  |
| **FlgB amino acid** | **Mutant number** | **Mutation^a^** | **Lac phenotype^b^** | **Motility^c^** | **ApR (PPBS plates)^d^** |
| 48 | AA39-48 -7 | E48V | ++++ | 100% | 100 |
| 48 | AA39-48-126 | E48V | ++++ | 100% | ND |
| 48 | AA39-48 1-3 | E48V | ++++ | 100% | ND |
| 50 | AA49-58 3 | K50* | - | 0% | 0 |
| 51 | AA49-58 28 | K51N | +/- | ND | ND |
| 51 | AA49-58 5 | R55L | +/- | ND | ND |
| 54 | AA49-58 1 | V54E | +/- | ND | ND |
| 58 | AA49-58 7 | E58* | - | 0% | ND |
| none | AA39-48-14 | none | ++++ | 100% | ND |
| none | AA39-48-33 | none | ++++ | 100% | ND |
| none | AA39-48-55 | none | ++++ | 100% | ND |
| none | AA39-48-75 | none | ++++ | 100% | ND |
| none | AA39-48-83 | none | ++++ | 100% | ND |
| none | AA39-48-92 | none | ++++ | 100% | ND |
| none | AA39-48-99 | none | ++++ | 100% | ND |
| none | AA39-48-125 | none | ++++ | 100% | ND |
| none | AA39-48-138 | none | ++++ | 100% | ND |
|  | WT | WT | ++++ | 100% | 75 |

| ^a^Mutations obtained in *flgB (**=STOP codon)  ^b^Strains carried *fljB5001*::MudJ Δ*hin*-*5718*::FRT alleles to assay for σ^28^-dependent class 3 flagellar gene transcription that was determined on Mac-Lac and TTC-lac indicator medium (37°C) (++++ : TTC-Lac white and ML dark red; +++ : TTC-Lac pink and ML dark red; ++ : TTC-Lac red and Mac-Lac red; +: TTC-Lac dark red and ML pink ; +/- : TTC-Lac dark red and Mac-Lac light pink; - : TTC-Lac dark red Mac-Lac white)  ^c^Motility phenotypes are given as percentage of WT motility at 37°C  ^d^*flgB* alleles were moved into a *flgB-bla* fusion expressed at the chromosomal *flg* locus. Ampicillin resistance levels were assayed on PPBS-Ap plates with varying ampicillin concentrations. ND: not determined. |
| --- |
