# Supplementary material for: Targeting early proximal-rod component substrate FlgB to FlhB for flagellar-type III secretion in Salmonella
Source: PLoS Genet. 2022 Jul 12;18(7):e1010313. doi: 10.1371/journal.pgen.1010313 (PMC9307174; doi:10.1371/journal.pgen.1010313)
Supplement: S3 Table — (DOCX) [file pgen.1010313.s007.docx]

| **S3 Table.** Randomization of codon F45 in *flgB* and class 3 activity phenotypes, motility and secretion assays |
| --- |

|  |  |  |  |  | **MIC assays^d^** | | |  |
| --- | --- | --- | --- | --- | --- | --- | --- | --- |
| **Colony number** | **F45^a^** | **Codon^a^** | **Lac**  **phenotype^b^** | **Motility^c^** | **1** | **2** | **3** | **Major Defect** |
| 5 | A | gcc | +/- | 5 | 25 | 25 | 25 | assembly |
| 30 | A | gct | +/- | 5 | 12 | 12 | 12 | secretion/assembly |
| 32 | A | gcg | +/- | 5 | 12 | 12 | 12 | secretion/assembly |
| 12--5 | A | gca | +/- | 5 | ND | ND | ND |  |
| 51 | V | gtt | ++ | 42 | 3 | 6 | 6 | secretion |
| 99 | V | gtg | + | 29 | 3 | 6 | 6 | secretion |
| 96-271 | V | gtc | ++ | 43 | 6 | 12 | 12 | secretion |
| 96-9 | V | gta | + | 22 | 3 | 3 | 3 | secretion |
| 22 | I | atc | ++ | 42 | 12 | 12 | 25 | secretion/assembly |
| 23 | I | att | ++ | 42 | 12 | 12 | 12 | secretion/assembly |
| 131 | I | ata | ++ | 43 | 3 | 6 | 6 | secretion |
| 31 | L | tta | ++ | 40 | ND | ND | ND | secretion |
| 36 | L | ctc | ++ | 40 | 6 | 6 | 12 | secretion |
| 143 | L | ttg | + | 30 | 3 | 6 | 6 | secretion |
| 96-13 | L | ctg | + | 21 | 6 | 6 | 6 | secretion |
| 96-21 | L | cta | + | 19 | 6 | 6 | 6 | secretion |
| 96-30 | L | ctt | + | 22 | 6 | 6 | 6 | secretion |
| 125 | M | atg | + | 31 | 3 | 6 | 6 | secretion |
| 129 | F | ttc | ++++ | 100 | 25 | 25 | 25 | WT |
| 144 | F | ttt | ++++ | 100 | 25 | 25 | 25 | WT |
| 18 | Y | tat | ++++ | 100 | 25 | 25 | 25 | WT |
| 113 | W | tgg | +++ | 60 | 12 | 12 | 12 | secretion |
| 15 | H | cac | + | 14 | 25 | 25 | 25 | assembly |
| 29 | H | cat | + | 14 | 12 | 12 | 12 | assembly |
| 35 | K | aag | - | 0 | 12 | 12 | 12 | secretion |
| 161 | K | aaa | - | 0 | 6 | 6 | 6 | secretion |
| 7 | R | cgt | - | 0 | ND | ND | ND | assembly |
| 26 | R | cga | - | 0 | ND | ND | ND | assembly |
| 28 | R | cgc | - | 0 | 6 | 6 | 6 | secretion |
| 39 | R | cgg | - | 0 | 3 | 6 | 6 | secretion |
| 12--2 | R | agg | - | 0 | ND | ND | ND |  |
| 12--3 | R | aga | - | 0 | ND | ND | ND |  |
| 20 | D | gac | - | 0 | ND | ND | ND |  |
| 126 | D | gat | - | 0 | 3 | 6 | 6 | secretion |
|  |  |  |  |  |  | | |  |
|  |  |  |  |  |  | | |  |
|  |  |  |  |  | **MIC assays^d^** | | |  |
| **Colony number** | **F45^a^** | **Codon^a^** | **Lac**  **phenotype^b^** | **Motility^c^** | **1** | **2** | **3** | **Major Defect** |
| 59 | E | gaa | - | 5 | 6 | 6 | 6 | secretion |
| 47 | N | aac | - | 0 | 6 | 12 | 12 | secretion |
| 12--1 | N | aat | - | 5 | ND | ND | ND |  |
| 13 | Q | cag | - | 0 | ND | ND | ND |  |
| 48 | Q | caa | - | 0 | 6 | 6 | 6 | secretion |
| 14 | S | tcg | - | 0 | 12 | 12 | 25 |  |
| 16 | S | agt | +/- | 5 | 12 | 12 | 12 | secretion/assembly |
| 40 | S | tct | +/- | 5 | 12 | 12 | 12 | secretion/assembly |
| 44 | S | tcc | +/- | 5 | 12 | 12 | 12 | secretion/assembly |
| 159 | S | tca | +/- | 2 | 12 | 12 | 12 | secretion/assembly |
| 12--4 | S | agc | +/- | 5 | ND | ND | ND |  |
| 122 | T | acg | + | 14 | 6 | 6 | 6 | secretion |
| 138 | T | act | + | 21 | 6 | 6 | 6 | secretion |
| 96-16 | T | acc | + | 19 | 6 | 12 | 12 | secretion |
| 96-266 | T | aca | + | 19 | 6 | 12 | 12 | secretion |
| 71 | C | tgt | + | 20 | 6 | 6 | 6 | secretion |
| 96-7 | C | tgc | + | 22 | 6 | 6 | 6 | secretion |
| 1 | G | ggg | - | 0 | 12 | 12 | 12 | assembly |
| 2 | G | gga | - | 0 | 12 | 12 | 12 | assembly |
| 3 | G | ggc | - | 0 | ND | ND | ND |  |
| 21 | G | ggt | - | 0 | ND | ND | ND | assembly |
| 10 | P | ccg | +/- | 5 | 12 | 12 | 25 | assembly |
| 25 | P | cct | +/- | 6 | 12 | 25 | 25 | assembly |
| 27 | P | ccc | +/- | 5 | 25 | 25 | 25 | assembly |
| 149 | P | cca | - | 0 | 6 | 6 | 6 | secretion/expression |
| 6 | stop | tga | - | 0 | 1.5 | 1.5 | 1.5 | expression |

^a^Amino acid and codon substitutions for amino acid 45 of FlgB

^b^Strains carried *fljB5001*::MudJ Δ*hin*-*5718*::FRT alleles to assay for σ^28^-dependent class 3 flagellar gene transcription which was determined on Mac-Lac and TTC-lac indicator medium (37°C) (++++ : TTC-Lac white and ML dark red; +++ : TTC-Lac pink and ML dark red; ++ : TTC-Lac red and Mac-Lac red; +: TTC-Lac dark red and ML pink ; +/- : TTC-Lac dark red and Mac-Lac light pink; - : TTC-Lac dark red Mac-Lac white)

^c^Motility phenotypes are given as percentage of WT motility at 37°C

^d^The MIC to Ap were measured in strains expressing *flgB-bla* with amino acid substitutions at codon 45 of *flgB* from the *araBAD* locus (P*_araBAD_*-*flgB-bla)* in strains deleted for the proximal rod components (Δ*flgBC* background). MIC assays were conducted on strains grown in the presence of arabinose. No arabinose added resulted in MIC’s of less than 1.5 μg/ml
