# Supplementary material for: Targeting early proximal-rod component substrate FlgB to FlhB for flagellar-type III secretion in Salmonella
Source: PLoS Genet. 2022 Jul 12;18(7):e1010313. doi: 10.1371/journal.pgen.1010313 (PMC9307174; doi:10.1371/journal.pgen.1010313)
Supplement: S4 Table — (DOCX) [file pgen.1010313.s008.docx]

**S4 Table.** Mutagenesis of the FlhB C-terminus surface exposed hydrophobic pocket (FlhB A286NNN A341VNN L344(VNN)) in a *fljB5001*::MudJ Δ*hin-5718*::FRT background to determine the effect of *flhB* mutations on HBB assembly.

| Mutants | Lac Phenotype | FlhB sequence | | |
| --- | --- | --- | --- | --- |
| **TetS pool**^a^ |  | A286 (gcg) | A341 (gcg) | L344  (ttg) |
| S1 | - | R (aga) | R (agg) | R (aga) |
| S5 | - | N (aat) | I (ata) | T (aca) |
| S6 | - | D (gac) | L (ctc) | G (ggg) |
| S7 | - | L (cta) | Q (caa) | E (gaa) |
| S16 | - | I (atc) | R (agg) | E (gag) |
| S3 | - | N (aac) | Q (caa) | H (cat) |
| S7 | - | V (gta) | L (ctg) | G (gga) |
| S8 | - | H (cac) | R (cgt) | T (act) |
| S11 | -/+ | S (agt) | R (aga) | R (cca) |
| S13 | -/+ | L (ctt) | K (aag) | G (ggg) |
| S9 | + | E (gaa) | L (ctc) | L (ctg) |
| S12 | + | E (gaa) | P (ggc) | I (atc) |
| **Lac+ pool^c^** |  |  |  |  |
| L3 | +/- | P (cca) | L (cta) | Q(caa) |
| L8 | +/- | P (ggt) | I (ata) | I(ata) |
| L9 | +/- | G (gga) | R (cga) | N(aat) |
| Lac 1 | +/- | V (gtg) | L (ctc) | L (ctg) |
| Lac 4 | +/- | A (gct) | A (gcc) | E (gaa) |
| Lac 5 | +/- | I (att) | I (atc) | I (ata) |
| L1 | ++ | E (gaa) | S (agc) | I (ata) |
| L2 | ++ | I (ata) | V (gta) | L (ctc) |
| L5 | ++ | V (gta) | T (aca) | L (ctt) |
| L6 | ++ | S (agc) | I (ata) | I (ata) |
| L7 | ++ | A (gcg) | Q (caa) | I (atc) |
| L10 | ++ | G (ggt) | I (att) | I (ata) |
| L16 | ++ | T (aca) | A (gca) | I (att) |
| Lac 2 | ++ | T (aca) | G (ggg) | L (ctg) |
| Lac 3 | ++ | A (gcg) | L (ctc) | L (ctt) |
| Lac 6 | ++ | V (gtt) | V (gta) | I (ata) |
| L4 | +++ | A (gcg) | V (gtt) | I (aat) |
| L11 | +++ | V (gtg) | G (ggg) | I (atc) |
| L13 | +++ | A (gct) | V (gta) | L (ctg) |
| L14 | +++ | A (gct) | A (gct) | L (ctc) |
| L15 | +++ | A (gca) | S (agt) | I (ata) |

^a^TetS pool corresponds a mixture of cells containing all the possible combinations of codons for FlhB A286NNN A341VNN L344(VNN). 12 colonies of this pool were sent for sequencing analysis to make sure the pool was varied.

^b^NCE pool corresponds to the Lac+ mutants (functional hook basal bodies) growing on NCE-lactose plates. Several mutants were purified and tested for their lac phenotypes on Mac-Lac and TTC-Lac plates.
