# Supplementary material for: Targeting early proximal-rod component substrate FlgB to FlhB for flagellar-type III secretion in Salmonella
Source: PLoS Genet. 2022 Jul 12;18(7):e1010313. doi: 10.1371/journal.pgen.1010313 (PMC9307174; doi:10.1371/journal.pgen.1010313)
Supplement: S5 Table — The FlhB A286NNN A341VNN L344VNN pool was transduced into several flgB mutant strains expressing flgB-bla alleles from ParaBAD, and screened for secretion on PPBS-Ara-Ap plates. (DOCX) [file pgen.1010313.s009.docx]

**S5 Table.** Searching for specific alleles in *flhB* that allow secretion of FlgB-Bla fusions expressed from P*_araBAD_* with amino acid substitutions at codon 45 of *flgB*. The FlhB A286NNN A341VNN L344(VNN) pool was transduced into several *flgB* allele strains expressing *flgB-bla* alleles from from P*_araBAD_*, and screened for secretion on PPBS-Ara-Ap plates.

| Recipient Strains | *flgB* allele | Number of PPBS-Ara-ApR mutants |
| --- | --- | --- |
| TH27380 | F45A | 5 |
| TH27381 | F45V | 0 |
| TH27382 | F45I | 0 |
| TH27383 | F45L | 1 |
| TH27384 | F45M | 0 |
| TH27385 | F45F | over 2000 |
| TH27386 | F45Y | over 500 |
| TH27428 | F45W | over 200 |
| TH27387 | F45H | 3 |
| TH27429 | F45K | 0 |
| TH27388 | F45R | 16 |
| TH27389 | F45D | 4 |
| TH27390 | F45E | 2 |
| TH27430 | F45N | 1 |
| TH27391 | F45Q | 0 |
| TH27392 | F45S | 7 |
| TH27431 | F45T | 1 |
| TH27393 | F45C | 2 |
| TH27394 | F45G | 1 |
| TH27395 | F45P | 0 |
