# Supplementary material for: Targeting early proximal-rod component substrate FlgB to FlhB for flagellar-type III secretion in Salmonella
Source: PLoS Genet. 2022 Jul 12;18(7):e1010313. doi: 10.1371/journal.pgen.1010313 (PMC9307174; doi:10.1371/journal.pgen.1010313)
Supplement: S6 Table — (DOCX) [file pgen.1010313.s010.docx]

**S6 Table.** List of strains used in this study

| **Strain** | **Genotype** |
| --- | --- |
| TH437 | wild type LT2 |
| TH3264 | *zec-7080*::Tn*10d*Tc |
| TH4725 | *flgI5085*::Tn*10d*Tc *flgK5267*::MudK |
| TH7365 | *fljB5001*::MudJ Δ*hin-5718*::FRT |
| TH8886 | Δ*pyrC3052*::FCF |
| TH13872 | *leu-1151*::Tn*10* Δ*hin-5718*::FRT *fljB5001*::MudJ |
| TH13954 | *STM1911*::Tn*10d*Tc |
| TH15317 | Δ*pyrC3052*::FCF Δ*flgN-L7736*::*tetRA* |
| TH23899 | *flgB8593*::*bla*(Bla (ΔSS) inserted before FlgB stop) |
| TH23902 | *flgC8595*::*bla*(Bla (ΔSS) inserted before FlgC stop) |
| TH23905 | *flgB8593*:*:bla* *flgB8596*::*tetRA*(ΔAA6-8) |
| TH23906 | *flgB8593*::*bla* *flgB8597*::*tetRA*(after AA28) |
| TH23907 | *flgB8593*::*bla* *flgB8598*::tetRA(after AA48) |
| TH23908 | *flgB8593*::*bla* *flgB8599*::tetRA(after AA68) |
| TH23909 | *flgB8593*::*bla* *flgB8600*::tetRA(after AA88) |
| TH23910 | *flgB8593*::*bla* *flgB8601*::tetRA(after AA108) |
| TH23926 | *flgB8603*::tetRA(after AA128) *flgB8593*::*bla* |
| TH23927 | *flgC8604*::tetRA(after AA4) *flgC8595*::*bla* |
| TH23928 | *flgC8605*::tetRA(after AA24) *flgC8595*::*bla* |
| TH23929 | *flgC8606*::tetRA(after AA44) *flgC8595*::*bla* |
| TH23930 | *flgC8607*::tetRA(after AA64) *flgC8595*::*bla* |
| TH23931 | *flgC8608*::tetRA(after AA84) *flgC8595*::*bla* |
| TH23932 | *flgC8609*::tetRA(after AA104) *flgC8595*::*bla* |
| TH23933 | *flgC8610*::tetRA(after AA124) *flgC8595*::*bla* |
| TH23990 | *flgB8615*(ΔAA2-8) *flgB8593*::*bla* |
| TH23991 | *flgB8616*(ΔAA9-18) *flgB8593*::*bla* |
| TH23992 | *flgB8617*(ΔAA19-28) *flgB8593*::*bla* |
| TH23993 | *flgB8618*(ΔAA29-38) *flgB8593*::*bla* |
| TH23994 | *flgB8619*(ΔAA39-48) *flgB8593*::*bla* |
| TH23995 | *flgB8620*(ΔAA49-58) *flgB8593*::*bla* |
| TH23996 | *flgB8621*(ΔAA59-68) *flgB8593*::*bla* |
| TH23997 | *flgB8622*(ΔAA69-78) *flgB8593*::*bla* |
| TH23998 | *flgB8623*(ΔAA79-88) *flgB8593*::*bla* |
| TH23999 | *flgB8624*(ΔAA89-98) *flgB8593*::*bla* |
| TH24000 | *flgB8625*(ΔAA99-108) *flgB8593*::*bla* |
| TH24001 | *flgB8626*(ΔAA109-118) *flgB8593*::*bla* |
| TH24002 | *flgB8627*(ΔAA119-128) *flgB8593*::*bla* |
| TH24003 | *flgB8628*(ΔAA129-138) *flgB8593*::*bla* |
| TH24004 | *flgC8629*(ΔAA2-6) *flgC8595*::*bla* |
| TH24005 | *flgC8630*(ΔAA5-14) *flgC8595*::*bla* |
| TH24006 | *flgC8631*(ΔAA15-24) *flgC8595*::*bla* |
| TH24007 | *flgC8632*(ΔAA25-34) *flgC8595*::*bla* |
| TH24008 | *flgC8633*(ΔAA35-44) *flgC8595*::*bla* |
| TH24009 | *flgC8634*(ΔAA45-54) *flgC8595*::*bla* |
| TH24010 | *flgC8635*(ΔAA55-64) *flgC8595*::*bla* |
| TH24011 | *flgC8636*(ΔAA65-74) *flgC8595*::*bla* |
| TH24012 | *flgC8637*(ΔAA75-84) *flgC8595*::*bla* |
| TH24013 | *flgC8638*(ΔAA85-94) *flgC8595*::*bla* |
| TH24014 | *flgC8639*(ΔAA95-104) *flgC8595*::*bla* |
| TH24015 | *flgC8640*(ΔAA105-114) *flgC8595*::*bla* |
| TH24016 | *flgC8641*(ΔAA115-124) *flgC8595*::*bla* |
| TH24017 | *flgC8642*(ΔAA125-134) *flgC8595*::*bla* |
| TH24147 | *flgF8649*::*bla*(Bla (ΔSS) inserted before FlgF stop) |
| TH24148 | *flgJ8650*::*bla*(Bla (ΔSS) inserted before FlgJ stop) |
| TH24149 | *flgG8651*::*bla*(Bla (ΔSS) inserted before FlgG stop) |
| TH24286 | *flgB8672*::tetRA(ΔAA6-8) *fljB5001*::MudJ Δ*hin*-*5718*::FRT |
| **Strain** | **Genotype** |
| TH24287 | *flgB8673*::tetRA(after AA28) *fljB5001*::MudJ Δ*hin*-*5718*::FRT |
| TH24288 | *flgB8674*::tetRA(after AA48) *fljB5001*::MudJ Δ*hin*-*5718*::FRT |
| TH24289 | *flgB8675*::tetRA(after AA68) *fljB5001*::MudJ Δ*hin*-*5718*::FRT |
| TH24290 | *flgB8676*::tetRA(after AA88) *fljB5001*::MudJ Δ*hin*-*5718*::FRT |
| TH24291 | *flgB8677*::tetRA(after AA108) *fljB5001*::MudJ Δ*hin*-*5718*::FRT |
| TH24292 | *flgB8678*::tetRA(after AA128) *fljB5001*::MudJ Δ*hin*-*5718*::FRT |
| TH24747 | *flgB8747*(F45L) *fljB5001*::MudJ Δ*hin*-*5718*::FRT |
| TH24748 | *flgB8748*(E48V) *fljB5001*::MudJ Δ*hin*-*5718*::FRT |
| TH24749 | *flgB8749*(F45L) *fljB5001*::MudJ Δ*hin*-*5718*::FRT |
| TH24750 | *flgB8750*(F45I) *fljB5001*::MudJ Δ*hin*-*5718*::FRT |
| TH24751 | *flgB8751*(I43S) *fljB5001*::MudJ Δ*hin*-*5718*::FRT |
| TH24752 | *flgB8752*(F45C) *fljB5001*::MudJ Δ*hin*-*5718*::FRT |
| TH24753 | *flgB8753*(D44Y) *fljB5001*::MudJ Δ*hin*-*5718*::FRT |
| TH24754 | *flgB8754*(D44G) *fljB5001*::MudJ Δ*hin*-*5718*::FRT |
| TH24755 | *flgB8755*(R41D) *fljB5001*::MudJ Δ*hin*-*5718*::FRT |
| TH24756 | *flgB8756*(F45V) *fljB5001*::MudJ Δ*hin*-*5718*::FRT |
| TH24757 | *flgB8757*(S47R) *fljB5001*::MudJ Δ*hin*-*5718*::FRT |
| TH24758 | *flgB8758*(D44V) *fljB5001*::MudJ Δ*hin*-*5718*::FRT |
| TH24759 | *flgB8759*(S47I) *fljB5001*::MudJ Δ*hin*-*5718*::FRT |
| TH24760 | *flgB8760*(A40S) *fljB5001*::MudJ Δ*hin*-*5718*::FRT |
| TH24761 | *flgB8761*(Q39L) *fljB5001*::MudJ Δ*hin*-*5718*::FRT |
| TH24762 | *flgB8762*(E48D) *fljB5001*::MudJ Δ*hin*-*5718*::FRT |
| TH24763 | *flgB8763*(I43F) *fljB5001*::MudJ Δ*hin*-*5718*::FRT |
| TH24764 | *flgB8764*(D42Y) *fljB5001*::MudJ Δ*hin*-*5718*::FRT |
| TH24765 | *flgB8747*(F45L) *flgB8593*::*bla* *fljB5001*::MudJ Δ*hin*-*5718*::FRT |
| TH24766 | *flgB8748*(E48V) *flgB8593*::*bla* *fljB5001*::MudJ Δ*hin*-*5718*::FRT |
| TH24767 | *flgB8749*(F45L) *flgB8593*::*bla* *fljB5001*::MudJ Δ*hin*-*5718*::FRT |
| TH24768 | *flgB8750*(F45I) *flgB8593*::*bla* *fljB5001*::MudJ Δ*hin*-*5718*::FRT |
| TH24769 | *flgB8751*(I43S) *flgB8593*::*bla* *fljB5001*::MudJ Δ*hin*-*5718*::FRT |
| TH24770 | *flgB8752*(F45C) *flgB8593*::*bla* *fljB5001*::MudJ Δ*hin*-*5718*::FRT |
| TH24771 | *flgB8753*(D44Y) *flgB8593*::*bla* *fljB5001*::MudJ Δ*hin*-*5718*::FRT |
| TH24772 | *flgB8754*(D44G) *flgB8593*::*bla* *fljB5001*::MudJ Δ*hin*-*5718*::FRT |
| TH24773 | *flgB8755*(R41D) *flgB8593*::*bla* *fljB5001*::MudJ Δ*hin*-*5718*::FRT |
| TH24774 | *flgB8756*(F45V) *flgB8593*::*bla* *fljB5001*::MudJ Δ*hin*-*5718*::FRT |
| TH24775 | *flgB8757*(S47R) *flgB8593*::*bla* *fljB5001*::MudJ Δ*hin*-*5718*::FRT |
| TH24776 | *flgB8758*(D44V) *flgB8593*::*bla* *fljB5001*::MudJ Δ*hin*-*5718*::FRT |
| TH24777 | *flgB8759*(S47I) *flgB8593*::*bla* *fljB5001*::MudJ Δ*hin*-*5718*::FRT |
| TH24778 | *flgB8760*(A40S) *flgB8593*::*bla* *fljB5001*::MudJ Δ*hin*-*5718*::FRT |
| TH24779 | *flgB8761*(Q39L) *flgB8593*::*bla* *fljB5001*::MudJ Δ*hin*-*5718*::FRT |
| TH24780 | *flgB8762*(E48D) *flgB8593*::*bla* *fljB5001*::MudJ Δ*hin*-*5718*::FRT |
| TH24781 | *flgB8763*(I43F) *flgB8593*::*bla* *fljB5001*::MudJ Δ*hin*-*5718*::FRT |
| TH24782 | *flgB8764*(D42Y) *flgB8593*::*bla* *fljB5001*::MudJ Δ*hin*-*5718*::FRT |
| TH25008 | pKD46/ Δ*araBAD2097*::5'UTR(*flgB*)-*flgB*::tetRA(after AA48)-*flgB*-*bla* Δ*flgBC*6557 |
| TH25371 | Δ*araBAD2096*::5'UTR(*flgB*)-*flgB*-*bla* Δ*pyrC3052*::FCF Δ*flgN-L7736*::*tetRA* *fljB5001*::MudJ Δ*hin- 5718*::FRT |
| TH25440 | Δ*araBAD2096*::5'UTR(*flgB*)-*flgB*-*bla* *fljB5001*::MudJ Δ*hin-5718*::FRT |
| TH25519 | Δ*araBAD2096*::5'UTR(*flgB*)-*flgB*-*bla* *fljB5001*::MudJ Δ*hin-5718*::FRT Δ*flgB-L8735* |
| TH25520 | Δ*araBAD2096*::5'UTR(*flgB*)-*flgB*-*bla* *fljB5001*::MudJ Δ*hin-5718*::FRT Δ*flgG*-*L2157* |
| TH25527 | Δ*araBAD2096*::5'UTR(*flgB*)-*flgB*-*bla* *fljB5001*::MudJ Δ*hin-5718*::FRT Δ*flgB-L8735 flhD8070 flhC8092 DrflM8403* |
| TH25528 | Δ*araBAD2096*::5'UTR(*flgB*)-*flgB*-*bla* *fljB5001*::MudJ Δ*hin-5718*::FRT Δ*flgG*-*L2157 flhD8070 flhC8092 DrflM8403* |
| TH25718 | Δ*araBAD2109*::5'UTR(*flgB*)-*flgB*(F45Y)-*bla* Δ*flgB-L8735* *flhD8070 flhC8092* *DrflM8403 fljB5001*::MudJ Δ*hin-5718*::FRT |
| TH25719 | Δ*araBAD2110*::5'UTR(*flgB*)-*flgB*(F45E)-*bla* Δ*flgB-L8735* *flhD8070 flhC8092* *DrflM8403 fljB5001*::MudJ Δ*hin-5718*::FRT |
| TH25720 | Δ*araBAD2111*::5'UTR(*flgB*)-*flgB*(F45H)-*bla* Δ*flgB-L8735* *flhD8070 flhC8092* *DrflM8403 fljB5001*::MudJ Δ*hin-5718*::FRT |
| TH26845 | pSIM5/*flgC8166*::tetRA(ΔAA49::tetRA) *fljB5001*::MudJ Δ*hin*-*5718*::FRT |
| TH27277 | Δ*araBAD2176*::(*flgB* 5'UTR)-*flgB*(F45A)-*bla* *fljB5001*::MudJ Δ*hin*-*5718*::FRT |
| **Strain** | **Genotype** |
| TH27278 | Δ*araBAD2177*::(*flgB* 5'UTR)-*flgB*(F45V)-*bla* *fljB5001*::MudJ Δ*hin*-*5718*::FRT |
| TH27279 | Δ*araBAD2178*::(*flgB* 5'UTR)-*flgB*(F45I)-*bla* *fljB5001*::MudJ Δ*hin*-*5718*::FRT |
| TH27280 | Δ*araBAD2179*::(*flgB* 5'UTR)-*flgB*(F45L)-*bla* *fljB5001*::MudJ Δ*hin*-*5718*::FRT |
| TH27281 | Δ*araBAD2180*::(*flgB* 5'UTR)-*flgB*(F45M)-*bla* *fljB5001*::MudJ Δ*hin*-*5718*::FRT |
| TH27282 | Δ*araBAD2181*::(*flgB* 5'UTR)-*flgB*(F45F)-*bla* *fljB5001*::MudJ Δ*hin*-*5718*::FRT |
| TH27283 | Δ*araBAD2182*::(*flgB* 5'UTR)-*flgB*(F45Y)-*bla* *fljB5001*::MudJ Δ*hin*-*5718*::FRT |
| TH27284 | Δ*araBAD2183*::(*flgB* 5'UTR)-*flgB*(F45H)-*bla* *fljB5001*::MudJ Δ*hin*-*5718*::FRT |
| TH27285 | Δ*araBAD2184*::(*flgB* 5'UTR)-*flgB*(F45R)-*bla* *fljB5001*::MudJ Δ*hin*-*5718*::FRT |
| TH27286 | Δ*araBAD2185*::(*flgB* 5'UTR)-*flgB*(F45D)-*bla* *fljB5001*::MudJ Δ*hin*-*5718*::FRT |
| TH27287 | Δ*araBAD2186*::(*flgB* 5'UTR)-*flgB*(F45E)-*bla* *fljB5001*::MudJ Δ*hin*-*5718*::FRT |
| TH27288 | Δ*araBAD2187*::(*flgB* 5'UTR)-*flgB*(F45Q)-*bla* *fljB5001*::MudJ Δ*hin*-*5718*::FRT |
| TH27289 | Δ*araBAD2188*::(*flgB* 5'UTR)-*flgB*(F45S)-*bla* *fljB5001*::MudJ Δ*hin*-*5718*::FRT |
| TH27290 | Δ*araBAD2189*::(*flgB* 5'UTR)-*flgB*(F45C)-*bla* *fljB5001*::MudJ Δ*hin*-*5718*::FRT |
| TH27291 | Δ*araBAD2190*::(*flgB* 5'UTR)-*flgB*(F45G)-*bla* *fljB5001*::MudJ Δ*hin*-*5718*::FRT |
| TH27292 | Δ*araBAD2191*::(*flgB* 5'UTR)-*flgB*(F45P)-*bla* *fljB5001*::MudJ Δ*hin*-*5718*::FRT |
| TH27299 | Δ*araBAD2192*::(*flgB* 5'UTR)-*flgB*(F45stop)-*bla* *fljB5001*::MudJ Δ*hin*-*5718*::FRT |
| TH27380 | Δ*araBAD2176*::(*flgB* 5'UTR)-*flgB*(F45A)-*bla* Δ*flhB8220*::tetRA *fljB5001*::MudJ Δ*hin*-*5718*::FRT |
| TH27381 | Δ*araBAD2177*::(*flgB* 5'UTR)-*flgB*(F45V)-*bla* Δ*flhB8220*::tetRA *fljB5001*::MudJ Δ*hin*-*5718*::FRT |
| TH27382 | Δ*araBAD2178*::(*flgB* 5'UTR)-*flgB*(F45I)-*bla* Δ*flhB8220*::tetRA *fljB5001*::MudJ Δ*hin*-*5718*::FRT |
| TH27383 | Δ*araBAD2179*::(flgB 5'UTR)-flgB(F45L)-*bla* Δ*flhB8220*::tetRA *fljB5001*::MudJ Δ*hin*-*5718*::FRT |
| TH27384 | Δ*araBAD2180*::(*flgB* 5'UTR)-*flgB*(F45M)-*bla* Δ*flhB8220*::tetRA *fljB5001*::MudJ Δ*hin*-*5718*::FRT |
| TH27385 | Δ*araBAD2181*::(*flgB* 5'UTR)-*flgB*(F45F)-*bla* Δ*flhB8220*::tetRA *fljB5001*::MudJ Δ*hin*-*5718*::FRT |
| TH27386 | Δ*araBAD2182*::(*flgB* 5'UTR)-*flgB*(F45Y)-*bla* Δ*flhB8220*::tetRA *fljB5001*::MudJ Δ*hin*-*5718*::FRT |
| TH27387 | Δ*araBAD2183*::(*flgB* 5'UTR)-*flgB*(F45H)-*bla* Δ*flhB8220*::tetRA *fljB5001*::MudJ Δ*hin*-*5718*::FRT |
| TH27388 | Δ*araBAD2184*::(*flgB* 5'UTR)-*flgB*(F45R)-*bla* Δ*flhB8220*::tetRA *fljB5001*::MudJ Δ*hin*-*5718*::FRT |
| TH27389 | Δ*araBAD2185*::(*flgB* 5'UTR)-*flgB*(F45D)-*bla* Δ*flhB8220*::tetRA *fljB5001*::MudJ Δ*hin*-*5718*::FRT |
| TH27390 | Δ*araBAD2186*::(*flgB* 5'UTR)-*flgB*(F45E)-*bla* Δ*flhB8220*::tetRA *fljB5001*::MudJ Δ*hin*-*5718*::FRT |
| TH27391 | Δ*araBAD2187*::(*flgB* 5'UTR)-*flgB*(F45Q)-*bla* Δ*flhB8220*::tetRA *fljB5001*::MudJ Δ*hin*-*5718*::FRT |
| TH27392 | Δ*araBAD2188*::(*flgB* 5'UTR)-*flgB*(F45S)-*bla* Δ*flhB8220*::tetRA *fljB5001*::MudJ Δ*hin*-*5718*::FRT |
| TH27393 | Δ*araBAD2189*::(*flgB* 5'UTR)-*flgB*(F45C)-*bla* Δ*flhB8220*::tetRA *fljB5001*::MudJ Δ*hin*-*5718*::FRT |
| TH27394 | Δ*araBAD2190*::(*flgB* 5'UTR)-*flgB*(F45G)-*bla* Δ*flhB8220*::tetRA *fljB5001*::MudJ Δ*hin*-*5718*::FRT |
| TH27395 | Δ*araBAD2191*::(*flgB* 5'UTR)-*flgB*(F45P)-*bla* Δ*flhB8220*::tetRA *fljB5001*::MudJ Δ*hin*-*5718*::FRT |
| TH27398 | Δ*araBAD2193*::(*flgB* 5'UTR)-*flgB*(F45W)-*bla* *fljB5001*::MudJ Δ*hin*-*5718*::FRT |
| TH27399 | Δ*araBAD2194*::(*flgB* 5'UTR)-*flgB*(F45K)-*bla* *fljB5001*::MudJ Δ*hin*-*5718*::FRT |
| TH27400 | Δ*araBAD2195*::(*flgB* 5'UTR)-*flgB*(F45N)-*bla* *fljB5001*::MudJ Δ*hin*-*5718*::FRT |
| TH27401 | Δ*araBAD2196*::(*flgB* 5'UTR)-*flgB*(F45T)-*bla* *fljB5001*::MudJ Δ*hin*-*5718*::FRT |
| TH27428 | Δ*araBAD2193*::(*flgB* 5'UTR)-*flgB*(F45W)-*bla* *flhB8220*::tetRA *fljB5001*::MudJ Δ*hin*-*5718*::FRT |
| TH27429 | Δ*araBAD2194*::(*flgB* 5'UTR)-*flgB*(F45K)-*bla* *flhB8220*::tetRA *fljB5001*::MudJ Δ*hin*-*5718*::FRT |
| TH27430 | Δ*araBAD2195*::(*flgB* 5'UTR)-*flgB*(F45N)-*bla* *flhB8220*::tetRA *fljB5001*::MudJ Δ*hin*-*5718*::FRT |
| TH27431 | Δ*araBAD2196*::(*flgB* 5'UTR)-*flgB*(F45T)-*bla* *flhB8220*::tetRA *fljB5001*::MudJ Δ*hin*-*5718*::FRT |
| TH27473 | Δ*araBAD2184*::(*flgB* 5'UTR)-*flgB*(F45R)-*bla* *flhB8916*::tetRA *fljB5001*::MudJ Δ*hin*-*5718*::FRT |
| TH27474 | *araBAD2186*::(*flgB* 5'UTR)-*flgB*(F45E)-*bla* *flhB8916*::tetRA *fljB5001*::MudJ Δ*hin*-*5718*::FRT |
| TH27519 | *araBAD2182*::(*flgB* 5'UTR)-*flgB*(F45Y)-*bla* *flhB9073* (A286A A341A L344Q) *fljB5001*::MudJ Δ*hin*-*5718*::FRT |
| TH27520 | *araBAD2182*::(*flgB* 5'UTR)-*flgB*(F45Y)-*bla* *flhB9074* (A286V A341A L344Q) *fljB5001*::MudJ Δ*hin*-*5718*::FRT |
| TH27521 | *araBAD2182*::(*flgB* 5'UTR)-*flgB*(F45Y)-*bla* *flhB9075* (A286V A341A L344H) *fljB5001*::MudJ Δ*hin*-*5718*::FRT |
| TH27522 | *araBAD2182*::(*flgB* 5'UTR)-*flgB*(F45Y)-*bla* *flhB9076* (A286V A341A L344V) *fljB5001*::MudJ Δ*hin*-*5718*::FRT |
| TH27523 | *araBAD2182*::(*flgB* 5'UTR)-*flgB*(F45Y)-*bla* *flhB9077* (A286P A341A L344H) *fljB5001*::MudJ Δ*hin*-*5718*::FRT |
| TH27524 | *araBAD2193*::(*flgB* 5'UTR)-*flgB*(F45W)-*bla* *flhB9078* (A286I A341A L344V) *fljB5001*::MudJ Δ*hin*-*5718*::FRT |
| TH27525 | *araBAD2193*::(*flgB* 5'UTR)-*flgB*(F45W)-*bla* *flhB9079* (A286Y A341A L344V) *fljB5001*::MudJ Δ*hin*-*5718*::FRT |
| TH27526 | *araBAD2193*::(*flgB* 5'UTR)-*flgB*(F45W)-*bla* *flhB9080* (A286V A341A L344V) *fljB5001*::MudJ Δ*hin*-*5718*::FRT |
| TH27527 | *araBAD2193*::(*flgB* 5'UTR)-*flgB*(F45W)-*bla* *flhB9081* (A286P A341A L344I) *fljB5001*::MudJ Δ*hin*-*5718*::FRT |
| TH27528 | *araBAD2179*::(*flgB* 5'UTR)-*flgB*(F45L)-*bla* *flhB9082* (A286P A341V L344L) *fljB5001*::MudJ Δ*hin*-*5718*::FRT |
| TH27529 | *araBAD2179*::(*flgB* 5'UTR)-*flgB*(F45L)-*bla* *flhB9083* (A286V A341T L344L) *fljB5001*::MudJ Δ*hin*-*5718*::FRT |
| TH27530 | *araBAD2179*::(*flgB* 5'UTR)-*flgB*(F45L)-*bla* *flhB9084* (A286T A341T L344M) *fljB5001*::MudJ Δ*hin*-*5718*::FRT |
| TH27531 | *araBAD2176*::(*flgB* 5'UTR)-*flgB*(F45A)-*bla* *flhB9085* (A286A A341R L344L) *fljB5001*::MudJ Δ*hin*-*5718*::FRT |
| TH27532 | *araBAD2176*::(*flgB* 5'UTR)-*flgB*(F45A)-*bla* *flhB9086* (A286Q A341I L344L) *fljB5001*::MudJ Δ*hin*-*5718*::FRT |
| TH27533 | *araBAD2188*::(*flgB* 5'UTR)-*flgB*(F45S)-*bla* *flhB9087* (A286A A341G L344A) *fljB5001*::MudJ Δ*hin*-*5718*::FRT |
| **Strain** | **Genotype** |
| TH27534 | *araBAD2188*::(*flgB* 5'UTR)-*flgB*(F45S)-*bla* *flhB9088* (A286T A341E L344L) *fljB5001*::MudJ Δ*hin*-*5718*::FRT |
| TH27535 | *araBAD2196*::(*flgB* 5'UTR)-*flgB*(F45T)-*bla* *flhB9089* (A286N A341E L344L) *fljB5001*::MudJ Δ*hin*-*5718*::FRT |
| TH27536 | *araBAD2189*::(*flgB* 5'UTR)-*flgB*(F45C)-*bla* *flhB9090* (A286G A341T L344I) *fljB5001*::MudJ Δ*hin*-*5718*::FRT |
| TH27537 | *araBAD2189*::(*flgB* 5'UTR)-*flgB*(F45C)-*bla* *flhB9091* (A286A A341R L344L) *fljB5001*::MudJ Δ*hin*-*5718*::FRT |
| TH27538 | *araBAD2184*::(*flgB* 5'UTR)-*flgB*(F45R)-*bla* *flhB9039* (A286A A341V L344E) *fljB5001*::MudJ Δ*hin*-*5718*::FRT |
| TH27545 | *araBAD2181*::(*flgB* 5'UTR)-*flgB*-*bla* *flhB9077* (A286P A341A L344H) *fljB5001*::MudJ Δ*hin*-*5718*::FRT |
| TH27546 | Δ*araBAD2198*::*flgC*-*bla* *fljB*5001::MudJ Δ*hin*-*5718*::FRT |
| TH27547 | Δ*araBAD2110*::5'UTR(*flgB*)-*flgB*(F45E)-*bla* Δ*flgB-L8735* *flhD8070 flhC8092* *DrflM8403 fljB5001*::MudJ Δ*hin-5718*::FRT *flhB9092* (L344R) |
| TH27548 | Δ*araBAD2184*::5'UTR(*flgB*)-*flgB*(F45R)-*bla* Δ*flgB-L8735* *flhD8070 flhC8092* *DrflM8403 fljB5001*::MudJ Δ*hin-5718*::FRT *flhB9093* (L344E) |
| TH27549 | Δ*araBAD2184*::5'UTR(*flgB*)-*flgB*(F45R)-*bla* *fljB5001*::MudJ Δ*hin-5718*::FRT *flhB9093* (L344E) |
| TH27562 | Δ*araBAD2186*::5'UTR(*flgB*)-*flgB*(F45E)-*bla* *fljB5001*::MudJ Δ*hin-5718*::FRT *flhB9092* (L344R) |
| CD 1 | *flgC9096* (F49G (ggc)) *fljB5001*::MudJ Δ*hin*-*5718*::FRT |
| CD 2 | *flgC9097* (F49L(cta)) *fljB5001*::MudJ Δ*hin*-*5718*::FRT |
| CD 3 | *flgC9098* (F49L(ctg)) *fljB5001*::MudJ Δ*hin*-*5718*::FRT |
| CD 4 | *flgC9099* (F49I (att)) *fljB5001*::MudJ Δ*hin*-*5718*::FRT |
| CD 5 | *flgC9100* (F49E (gaa)) *fljB5001*::MudJ Δ*hin*-*5718*::FRT |
| CD 6 | *flgC9101* (F49S (tcc)) *fljB5001*::MudJ Δ*hin*-*5718*::FRT |
| CD 7 | *flgC9102* (F49F (ttt)) *fljB5001*::MudJ Δ*hin*-*5718*::FRT |
| CD 8 | *flgC9103* (F49S (tcg)) *fljB5001*::MudJ Δ*hin*-*5718*::FRT |
| CD 9 | *flgC9104* (F49H (cac)) *fljB5001*::MudJ Δ*hin*-*5718*::FRT |
| CD 10 | *flgC9105* (F49Q (caa)) *fljB5001*::MudJ Δ*hin*-*5718*::FRT |
| CD 11 | *flgC9106* (F49A (gca)) *fljB5001*::MudJ Δ*hin*-*5718*::FRT |
| CD 12 | *flgC9107* (F49S (agt)) *fljB5001*::MudJ Δ*hin*-*5718*::FRT |
| CD 13 | *flgC9108* (F49D (gac)) *fljB5001*::MudJ Δ*hin*-*5718*::FRT |
| CD 14 | *flgC9109* (F49P (cct)) *fljB5001*::MudJ Δ*hin*-*5718*::FRT |
| CD 15 | *flgC9110* (F49N (aat)) *fljB5001*::MudJ Δ*hin*-*5718*::FRT |
| CD 16 | *flgC9111* (F49K (aaa)) *fljB5001*::MudJ Δ*hin*-*5718*::FRT |
| CD 17 | *flgC9112* (F49V (gta)) *fljB5001*::MudJ Δ*hin*-*5718*::FRT |
| CD 18 | *flgC9113* (F49R (cga)) *fljB5001*::MudJ Δ*hin*-*5718*::FRT |
| CD 19 | *flgC9114* (F49I (ata)) *fljB5001*::MudJ Δ*hin*-*5718*::FRT |
| CD 20 | *flgC9115* (F49STOP (taa)) *fljB5001*::MudJ Δ*hin*-*5718*::FRT |
| CD 21 | *flgC9116* (F49Y (tat)) *fljB5001*::MudJ Δ*hin*-*5718*::FRT |
| CD 22 | *flgC9117* (F49R (agg)) *fljB5001*::MudJ Δ*hin*-*5718*::FRT |
| CD 23 | *flgC9118* (F49L (ctc)) *fljB5001*::MudJ Δ*hin*-*5718*::FRT |
| CD 24 | *flgC9119* (F49E (gag)) *fljB5001*::MudJ Δ*hin*-*5718*::FRT |
| CD 25 | *flgC9120* (F49K (aag)) *fljB5001*::MudJ Δ*hin*-*5718*::FRT |
| CD 26 | *flgC9121* (F49R (cgt)) *fljB5001*::MudJ Δ*hin*-*5718*::FRT |
| CD 27 | *flgC9122* (F49Y (tac)) *fljB5001*::MudJ Δ*hin*-*5718*::FRT |
| CD 28 | *flgC* *9123* (F49T (acc)) *fljB5001*::MudJ Δ*hin*-*5718*::FRT |
| CD 29 | *flgC9124* (F49A (gcg)) *fljB5001*::MudJ Δ*hin*-*5718*::FRT |
| CD 30 | *flgC9125* (F49P (ccg)) *fljB5001*::MudJ Δ*hin*-*5718*::FRT |
| CD 31 | *flgC9126* (F49S (tct)) *fljB5001*::MudJ Δ*hin*-*5718*::FRT |
| CD 32 | *flgC9127* (F49G (ggt)) *fljB5001*::MudJ Δ*hin*-*5718*::FRT |
| CD 33 | *flgC9128* (F49A (gct)) *fljB5001*::*MudJ* Δ*hin*-*5718*::FRT |
| CD 34 | *flgC* *9129* (F49STOP (tag)) *fljB5001*::MudJ Δ*hin*-*5718*::FRT |
| CD 35 | *flgC9130* (F49P (ccc)) *fljB5001*::MudJ Δ*hin*-*5718*::FRT |
| CD 36 | *flgC9130* (F49R (cgc)) *fljB5001*::MudJ Δ*hin*-*5718*::FRT |
| CD 37 | *flgC* *9130* (F49C (tgc)) *fljB5001*::MudJ Δ*hin*-*5718*::FRT |
| CD 38 | *flgC9130* (F49T (aca)) *fljB5001*::MudJ Δ*hin*-*5718*::FRT |
| CD 39 | *flgC9130* (F49M (atg)) *fljB5001*::MudJ Δ*hin*-*5718*::FRT |
| CD 40 | *flgC* *9130* (F49W (tgg)) *fljB5001*::MudJ Δ*hin*-*5718*::FRT |
| CD 41 | *flgC* *9130* (F49S (agc)) *fljB5001*::MudJ Δ*hin*-*5718*::FRT |
| CD 42 | *flgC9130* (F49P (cca)) *fljB5001*::MudJ Δ*hin*-*5718*::FRT |
| CD 43 | Δ*araBAD2199*::*flgC* (F49F)-*bla* Δ*flgBC6557* |
|  |  |
| **Strain** | **Genotype** |
| CD44 | Δ*araBAD2200*::*flgC* (F49L)-*bla* Δ*flgBC6557* |
| CD 45 | Δ*araBAD2201*::*flgC* (F49I)-*bla* Δ*flgBC6557* |
| CD 46 | Δ*araBAD2202*::*flgC* (F49D)-*bla* Δ*flgBC6557* |
| CD 47 | Δ*araBAD2203*::*flgC* (F49K)-*bla* Δ*flgBC6557* |
| CD 48 | Δ*araBAD2204*::*flgC* (F49E)-*bla* Δ*flgBC6557* |
| CD 49 | Δ*araBAD2205*::*flgC* (F49R)-*bla* Δ*flgBC6557* |
| CD 50 | Δ*araBAD2206*::*flgC* (F49S)-*bla* Δ*flgBC6557* |
| CD 51 | Δ*araBAD2207*::*flgC* (F49G)-*bla* Δ*flgBC6557* |
| CD 52 | Δ*araBAD2208*::*flgC* (F49P)-*bla* Δ*flgBC6557* |
| CD 53 | Δ*araBAD2209*::*flgC* (F49V)-*bla* Δ*flgBC6557* |
| CD 56 | *flgC9131* (F49V (gtg)) *fljB5001*::MudJ Δ*hin*-5718::FRT |
| CD 57 | *flgC9132* (F49V (gtc)) *fljB5001*::MudJ Δ*hin*-5718::FRT |
| CD 58 | *flgC9133* (F49V (gta)) *fljB5001*::MudJ Δ*hin*-5718::FRT |
| CD 59 | *flgC9134* (F49V (gtt)) *fljB5001*::MudJ Δ*hin*-5718::FRT |
| CD 60 | *fljB5001*::MudJ Δ*hin*-*5718*::FRT *flhB* (286VNN 341VNN 344VNN) TetS POOL (at least 150,000 colonies) |
| CD 62 | *fljB5001*::MudJ Δ*hin*-*5718*::FRT *flhB* (286VNN 341VNN 344VNN) NCE Lac^+^ POOL |
| CD 63 | Δ*araBAD2210*::*flgC* (F49A)-*bla* Δ*flgBC6557* |
| CD 64 | Δ*araBAD2211*::*flgC* (F49M)-*bla* Δ*flgBC6557* |
| CD 65 | Δ*araBAD2212*::*flgC* (F49Y)-*bla* Δ*flgBC6557* |
| CD 66 | Δ*araBAD2213*::*flgC* (F49W)-*bla* Δ*flgBC6557* |
| CD 67 | Δ*araBAD2214*::*flgC* (F49H)-*bla* Δ*flgBC6557* |
| CD 68 | Δ*araBAD2215*::*flgC* (F49N)-*bla* Δ*flgBC6557* |
| CD 69 | Δ*araBAD2216*::*flgC* (F49T)-*bla* Δ*flgBC6557* |
| CD 70 | Δ*araBAD2217*::*flgC* (F49C)-*bla* Δ*flgBC6557* |
| CD 71 | Δ*araBAD2218*::*flgC* (F49V)-*bla* Δ*flgBC6557* |
| CD 72 | Δ*araBAD2219*::*flgC* (F49Q)-*bla* Δ*flgBC6557* |
| CD 73 | *fljB5001*::*MudJ* Δ*hin*-*5718*::FRT *flhB* (286VNN 341VNN 344VNN) Lac^+^ POOL refined in liquid NCE-Lac |
| CD 74 | *fljB5001*::MudJ Δ*hin*-*5718*::FRT *flhB* (286VNN 341VNN 344VNN) NCE Lac^+^ POOL refined on plates |
| CD 75 | *fljB5001*::MudJ Δ*hin*-*5718*::FRT *flhB* (286VNN 341VNN 344VNN) 1000 colonies Lac^+++^ POOL |
| TH27591 | Δ*araBAD2210::flgC(F49A)-bla fljB5001::MudJ* Δ*hin-5718::FRT* |
| TH27592 | Δ*araBAD2218::flgC(F49V)-bla fljB5001::MudJ* Δ*hin-5718::FRT* |
| TH27593 | Δ*araBAD2201::flgC(F49I)-bla fljB5001::MudJ*  Δ*hin-5718::FRT* |
| TH27594 | Δ*araBAD2200::flgC(F49AL)-bla fljB5001::MudJ* Δ*hin-5718::FRT* |
| TH27595 | Δ*araBAD2211::flgC(F49M)-bla fljB5001::MudJ* Δ*hin-5718::FRT* |
| TH27596 | Δ*araBAD2212::flgC(F49Y)-bla fljB5001::MudJ* Δ*hin-5718::FRT* |
| TH27597 | Δ*araBAD2213::flgC(F49W)-bla fljB5001::MudJ* Δ*hin-5718::FRT* |
| TH27598 | Δ*araBAD2214::flgC(F49H)-bla fljB5001::MudJ* Δ*hin-5718::FRT* |
| TH27599 | Δ*araBAD2203::flgC(F49K)-bla fljB5001::MudJ* Δ*hin-5718::FRT* |
| TH27600 | Δ*araBAD2205::flgC(F49R)-bla fljB5001::MudJ* Δ*hin-5718::FRT* |
| TH27601 | Δ*araBAD2202::flgC(F49D)-bla fljB5001::MudJ* Δ*hin-5718::FRT* |
| TH27602 | Δ*araBAD2204::flgC(F49E)-bla fljB5001::MudJ* Δ*hin-5718::FRT* |
| TH27603 | Δ*araBAD2215::flgC(F49N)-bla fljB5001::MudJ* Δ*hin-5718::FRT* |
| TH27604 | Δ*araBAD2219::flgC(F49Q)-bla fljB5001::MudJ* Δ*hin-5718::FRT* |
| TH27605 | Δ*araBAD2206::flgC(F49S)-bla fljB5001::MudJ* Δ*hin-5718::FRT* |
| TH27606 | Δ*araBAD2216::flgC(F49T)-bla fljB5001::MudJ* Δ*hin-5718::FRT* |
| TH27607 | Δ*araBAD2217::flgC(F49C)-bla fljB5001::MudJ* Δ*hin-5718::FRT* |
| TH27608 | Δ*araBAD2207::flgC(F49G)-bla fljB5001::MudJ* Δ*hin-5718::FRT* |
| TH27609 | Δ*araBAD2208::flgC(F49P)-bla fljB5001::MudJ* Δ*hin-5718::FRT* |
