# Supplementary material for: Targeting early proximal-rod component substrate FlgB to FlhB for flagellar-type III secretion in Salmonella
Source: PLoS Genet. 2022 Jul 12;18(7):e1010313. doi: 10.1371/journal.pgen.1010313 (PMC9307174; doi:10.1371/journal.pgen.1010313)
Supplement: S7 Table — (DOCX) [file pgen.1010313.s011.docx]

**S7 Table.** List of primers used in this study

**No Name Sequence (5’----3’)**

1301-flgFtetA tttggcgatccataatgaactgatcataaaatgtcctgtactaagcacttgtctcctg

1750-flgCEbla gatcttcagcatcttttactttcaccagcgtttctgggtgctggcctaatgtcagcgttt

1809-flgFGtetRA3 tcagggagccggtggcgtatcccccaccggcggccccttactaagcacttgtctcctg

3822-FlgCAraB TACTGTTTCTCCATACCTGTTTTTCTGGATGGAGTAAGACGATGGCGCTGTTAAACATTT

4193-Flgkendblarev tcgttgttatcggcagcgactacgtggacttgagcaatttaccaatgcttaatcagtgag

6489-flgG_tetR260 gtatcgacgaccgatcagatgctgcagaaactgacgcaactcttaagacccactttcaca

7793-flgB-bla-fw agccaactcaaaggcatgatgaatgtgctacagggaggaaacCACCCAGAAACGCTGGTGA

7794-flgB-bla-rev gatccggcaatatcaaaaatgtttaacagcgccacgaattaCCAATGCTTAATCAGTGAGGC

7795-FlgC-bla-fw accgtaaaaagcatgatgcttaaaacgctgacattaggccagCACCCAGAAACGCTGGTGA

7796-FlgC-blarev gggtcattcatatttacggcaatagacatacgcgcctcctttaCCAATGCTTAATCAGTGAGGC

7824-flgB-AA8-tetA acgttgcgcgcgcagatttagcgcttcctgctgaaatcgtaaCTAAGCACTTGTCTCCTG

7825-flgB-AA28-tetR gctaaatctgcgcgcgcaacgtcaggaaatattagcggcgTTAAGACCCACTTTCACATT

7826-flgB-AA28-tetA atcgcgcgcctgataccccggcgtatcggcattggcgatattCTAAGCACTTGTCTCCTG

7827-flgB-AA48-tetR gccggggtatcaggcgcgcgatattgattttgccagtgagTTAAGACCCACTTTCACATT

7828-flgB-AA48-tetA gccgccggtttcttcccgtccacgcaccatcaccttttttaaCTAAGCACTTGTCTCCTG

7829-flgB-AA68-tetR tggacgggaagaaaccggcggcgtcgcgttaacgttgactTTAAGACCCACTTTCACATT

7830-flgB-AA68-tetA tgcgggagaagagaccgcctgggcgggaatatggtgagaggaCTAAGCACTTGTCTCCTG

7831-flgB-AA88-tetR ccaggcggtctcttctcccgcagtggatctgctttatcgcTTAAGACCCACTTTCACATT

7832-flgB-AA88-tetA catatctacggtattaccatccaaagaaggctgatcgggtacCTAAGCACTTGTCTCCTG

7833-flgB-AA108-tetR ggatggtaataccgtagatatggacagggaacgtacgcagTTAAGACCCACTTTCACATT

7834-flgB-AA108-tetA aacggtaagccccatctgatatttgagactgttatccgcaaaCTAAGCACTTGTCTCCTG

7835-flgB-AA128-tetR atatcagatggggcttaccgttctgggtagccaactcaaaTTAAGACCCACTTTCACATT

7836-flgB-AA128tetA tttctgggtggtttcctccctgtagcacattcatcatgcc CTAAGCACTTGTCTCCTG

7837-flgC-AA4tetR gaatgtgctacagggaggaaacTAAttcGTGgcgctgtta TTAAGACCCACTTTCACATT

7838-flgC-AA4tetA actgtgcggcaagcgccgatccggcaatatcaaaaatgtt CTAAGCACTTGTCTCCTG

7839-flgC-AA24tetR atcggcgcttgccgcacagtccaaacggttgaacgttgcgTTAAGACCCACTTTCACATT

7840-flgC-AA24tetA ccgggccggtgacgctatccgcattcgccaggttactggcCTAAGCACTTGTCTCCTG

7841-flgC-AA44tetR ggatagcgtcaccggcccggacggacagccttatcgcgcc TTAAGACCCACTTTCACATT

7842-flgC-AA44tetA cttgacccggcgcggcgtccacctgaaaaaccacctgttt CTAAGCACTTGTCTCCTG

7843-flgC-AA64tetR ggacgccgcgccgggtcaagccactggcggggtaaaggtc TTAAGACCCACTTTCACATT

7844-flgC-AA64tetA aaaccagcttttccggtgcctgactttcaatcacgctggc CTAAGCACTTGTCTCCTG

7845-flgC-AA84tetR ggcaccggaaaagctggtttatgagccaggcaatccgctg TTAAGACCCACTTTCACATT

7846-flgC-AA84tetA catcgacgttgggcattttgacgtaaccattagcgtccgc CTAAGCACTTGTCTCCTG

7847-flgC-AA104tetR caaaatgcccaacgtcgatgtggtcggcgaaatggtcaac TTAAGACCCACTTTCACATT

7848-flgC-AA104tetA cttcgatatttgcctgatagctgcgcgaggctgacatcgt CTAAGCACTTGTCTCCTG

7849-flgC-AA124tetR ctatcaggcaaatatcgaagtcctgaataccgtaaaaagc TTAAGACCCACTTTCACATT

7850-flgC-AA124tetA tttctgggtg ctggcctaatgtcagcgttttaagcatcat CTAAGCACTTGTCTCCTG

7851-DflgB2-8fw aagctgtcggctgaattttgccatttgcggaggagatATG ttacgatttcagcaggaa

7852-DflgB2-8rv acgttgcgcgcgcagatttagcgcttcctgctgaaatcgtaa

7853-DflgB9-18fw tgaattttgccatttgcggaggagatATGctcgacaggctcgatgccgcc

7854-DflgB9-18rv tggcgatattcgccgctaatatttcctgacgttgcgcgcg GGCGGCATCGAGCCTGTC

7855-DflgB19-28fw cgatgccgccttacgatttcagcaggaagcgctaaatctg aatatcgccaatgccgat

7856-DflgB19-28rev tatcgcgcgcctgataccccggcgtatcggcattggcgatatt

7857-DflgB29-38fw gctaaatctgcgcgcgcaacgtcaggaaatattagcggcg caggcgcgcgatattgat

7858-DflgB29-38fw tcaccttttttaactcactggcaaaatcaatatcgcgcgcctg

7859-DflgB39-48fw attagcggcgaatatcgccaatgccgatacgccggggtat ttaaaaaaggtgatggtg

7860-DflgB39-48rv cgccgccggtttcttcccgtccacgcaccatcaccttttttaa

7861 DflgB49-58fw gccggggtatcaggcgcgcgatattgattttgccagtgag gaaaccggcggcgtcgcg

7862-DflgB49-58rv tatggtgagaggaagtcaacgttaacgcgacgccgccggtttc

7863-DflgB59-68fw tgccagtgagttaaaaaaggtgatggtgcgtggacgggaaTCCTCTCACCATATTCCC

7864-DflgB59-68rev tgcgggagaagagaccgcctgggcgggaatatggtgagagga

7865-DflgB69-78fw tggacgggaagaaaccggcggcgtcgcgttaacgttgact tcttctcccgcagtggat

7866-DflgB69-78rev ctgatcgggtacgcgataaagcagatccactgcgggagaaga

7867-DflgB79-88fw aacgttgacttcctctcaccatattcccgcccaggcggtc gtacccgatcagccttct

7868-DflgB79-88rev catatctacggtattaccatccaaagaaggctgatcgggtac

7869-DflgB89-98fw ccaggcggtctcttctcccgcagtggatctgctttatcgc accgtagatatggacagg

7870-DflgB89-98rev ctgttatccgcaaactgcgtacgttccctgtccatatctacggt

7871-DflgB99-108fw gctttatcgcgtacccgatcagccttctttggatggtaat tttgcggataacagtctc

**No Name Sequence (5’----3’)**

7872-DflgB99-108rev gaacggtaagccccatctgatatttgagactgttatccgcaaa

7873-DflgB109-118fw ggatggtaataccgtagatatggacagggaacgtacgcag gggcttaccgttctgggt

7874-DflgB109-118rev cattcatcatgcctttgagttggctacccagaacggtaagccc

7875-DflgB119-128fw acgtacgcagtttgcggataacagtctcaaatatcagatg ggcatgatgaatgtgcta

7876-DflgB119-128rv cgtttctgggtg gtttcctccctgtagcacattcatcatgcc

7877-DflgB120-138fw aaatatcagatggggcttaccgttctgggtagccaactcaaa

7878-DflgB120-138rev gatcttcagcatcttttactttcaccagcgtttctgggtg tttgagttggctacccag

77896-FlgC-D2-6fw aggcatgatgaatgtgctacagggaggaaactaattcgtgTTTGATATTGCCGGATCG

7897-flgC-D2-6rev acgttcaaccgtttggactgtgcggcaagcgccgatccggcaatatcaaa

7898-flgC-D5-14fw gaatgtgctacagggaggaaacTAAttcGTGgcgctgttaGCCGCACAGTCCAAACG

7899-flgC-D5-14rv gcattcgccaggttactggccgcaacgttcaaccgtttggactgtgcggc

7900-flgC-D15-24fw ggcgctgttaaacatttttgatattgccggatcggcgctt GCCAGTAACCTGGCGAATG

7901-flgC-D15-24rv ggctgtccgtccgggccggtgacgctatccgcattcgccaggttactggc

7902-flgC-D25-34fw atcggcgcttgccgcacagtccaaacggttgaacgttgcg ACCGGCCCGGACGGACAG

7903-flgC-D25-34rv acctgaaaaaccacctgtttggcgcgataaggctgtccgtccgggccggt

7904-flgC-D35-44fw gaacgttgcggccagtaacctggcgaatgcggatagcgtc AAACAGGTGGTTTTTCAGG

7905-flgC-D35-44rv ccgccagtggcttgacccggcgcggcgtccacctgaaaaaccacctgttt

7906-flgC-D45-54fw ggatagcgtcaccggcccggacggacagccttatcgcgccCCGGGTCAAGCCACTGGC

7907-flgC-D45-54rv tgactttcaatcacgctggcgacctttaccccgccagtggcttgacccgg

7908-flgC-D55-64fw ttatcgcgccaaacaggtggtttttcaggtggacgccgcgGCCAGCGTGATTGAAAGT

7909-flgC-D55-64rv cctggctcataaaccagcttttccggtgcctgactttcaatcacgctggc

7910-flgC-D65-74fw ggacgccgcgccgggtcaagccactggcggggtaaaggtcAAGCTGGTTTATGAGCCA

7911-flgC-D65-74rv acgtaaccattagcgtccgccagcggattgcctggctcataaaccagctt

7912-flgC-D75-84fw ggtaaaggtcgccagcgtgattgaaagtcaggcaccggaaGCGGACGCTAATGGTTAC

7913-flgC-D75-84rv tcgccgaccacatcgacgttgggcattttgacgtaaccattagcgtccgc

7914-flgC-D85-94fw ggcaccggaaaagctggtttatgagccaggcaatccgctgAACGTCGATGTGGTCGGC

7915-flgC-D85-94rv ctgcgcgaggctgacatcgtgttgaccatttcgccgaccacatcgacgtt

7916-flgC-D95-104fw caatccgctggcggacgctaatggttacgtcaaaatgcccACGATGTCAGCCTCGCG

7917-flgC-D95-104rv gtattcaggacttcgatatttgcctgatagctgcgcgaggctgacatcgt

7918-flgC-D105-114fw caaaatgcccaacgtcgatgtggtcggcgaaatggtcaac AATATCGAAGTCCTGAATAC

7919-flgC-D105-114rv gtcagcgttttaagcatcatgctttttacggtattcaggacttcgatatt

7920-flgC-115-124fw aatggtcaacacgatgtcagcctcgcgcagctatcaggca ATGATGCTTAAAACGCTG

7921-flgC115-124rv- ttcaccagcgtttctgggtg ctggcctaatgtcagcgttttaagcatcat

7922-flgC-D125-134fw cctcgcgcagctatcaggcaaatatcgaagtcctgaataccgtaaaaagc

7923-flgC-D125-134rv gatcttcagcatcttttactttcaccagcgtttctgggtg GCTTTTTACGGTATTCAG

7970-flgF-stopbla tttggcgatccataatgaactgatcataaaatgtcctgtattaccaatgcttaatcagtgaggc

7971-flgG-stopbla atcgacgaccgatcagatgctgcagaaactgacgcaactccacccagaaacgctggtga

7972-flgG-stopbla tcagggagccggtggcgtatcccccaccggcggccccttaccaatgcttaatcagtga

8096-flgB39-fw tcaggaaatattagcggcgaatatcgccaatgccgatacg

8097-flgB48-rev aacgcgacgccgccggtttcttcccgtccacgcaccatcaccttt

8098-flgB49fw gccaatgccgatacgccggggtatcaggcgcgcgatattgattttgccagtgag

8099-flgB-58-rev ggaatatggtgagaggaagtcaacgttaacgcgac

| 8100-flgBdopedAA39-48 | atcgccaatgccgatacgccggggtatCAGGCGCGCGATATTGATTTTGCCAGTGAGttaaa  aaaggtgatggtgcgtgg |
| --- | --- |
| 8101-flgB-doped-aa49-58 | gatattgattttgccagtgagTTA AAA AAG GTG ATG GTG CGT GGA CGG GAAgaaaccggcggcgtcgcgttaacgttgact |

8316-flgB-F45NNN atcgccaatgccgatacgccggggtatcaggcgcgcgatattgat NNN gccagtgagttaaaaaaggtgatggtgcgtgg

9218-FlgCNNN49rev ttaccccgccagtggcttgacccggcgcggcgtccacctgNNNaaccacctgtttggcgcg

9219-FlgCNNN49fill tagcgtcaccggcccggacggacagccttatcgcgccaaacaggtggtt

9631-flhB3602-illu-fw ACACTCTTTCCCTACACGACGCTCTTCCGATCTccgcatgatggaagatgtg

9632-flhB8907illu-rv GACTGGAGTTCAGACGTGTGCTCTTCCGATCTaggttctcaggttgtgga
